# Integrated SMRT and Illumina Sequencing Provide New Insights into Crocin Biosynthesis of *Gardenia jasminoides*

**DOI:** 10.3390/ijms23116321

**Published:** 2022-06-05

**Authors:** Tengfei Shen, Yongjie Zheng, Qian Liu, Caihui Chen, Lili Huang, Shaoyong Deng, Meng Xu, Chunxia Yang

**Affiliations:** 1 Jiangxi Academy of Forestry, Nanchang 330032, China; tengfeishen@njfu.edu.cn (T.S.); zyj_bio2015@163.com (Y.Z.); 18670223641@163.com (Q.L.); hll6571@163.com (L.H.); jxforestry@163.com (S.D.); 2 Co-Innovation Center for Sustainable Forestry in Southern China, Nanjing Forestry University, 159 Longpan Road, Nanjing 210037, China; 3 Institute of Biological Resources, Jiangxi Academy of Sciences, Nanchang 330096, China; chencaihui0110@163.com

**Keywords:** *Gardenia jasminoides*, SMRT sequencing, RNA-seq, crocin content, crocin biosynthesis

## Abstract

Crocins are valuable bioactive components of gardenia fruit, and their biosynthesis and accumulation have attracted widespread interest. Studies have investigated the biosynthesis and accumulation of crocin based on Illumina sequencing, but there is a lack of reports based on full-length transcriptome sequencing. Utilising SMRT sequencing and high-performance liquid chromatography (HPLC), we explored crocin biosynthesis and accumulation in the fruit of *Gardenia jasminoides*. HPLC analysis showed that crocins specifically exist in fruit and that the content of crocins increases gradually during fruit development. SMRT sequencing generated 46,715 high-quality full-length isoforms, including 5230 novel isoforms that are not present in the *G. jasminoides* genome. Furthermore, a total of 46 genes and 91 lncRNAs were involved in the biosynthesis and accumulation of crocin. The qRT-PCR indicated that genes involved in crocin biosynthesis reached a peak in the NOV stage. These findings contributed to our understanding of crocin biosynthesis and accumulation.

## 1. Introduction

*Gardenia jasminoides* Ellis is an evergreen flowering plant in the Rubiaceae family with great medicinal and edible value, as it exhibits liver protection, anti-inflammatory, heat-clearing, detoxicating, and other effects [1,2,3]. As the main bioactive constituent in *G. jasminoides* fruit, crocin is an apocarotenoid compound that is involved in important commercial applications as textile colourants and histochemical stains, as an antiangiogenic and neuroprotective agent, and in the prevention of tumour cell proliferation in vitro [4,5,6,7]. Due to their complicated chemical and structural properties, crocins cannot be synthesised chemically, and there has been burgeoning industrial interest in their biotechnological production.

Apocarotenoids are derived from carotenoids by oxidative cleavage. The reported pathway for crocin biosynthesis begins with the cleavage of zeaxanthin in *Crocus sativus*, which is catalysed by the enzyme carotenoid cleavage dioxygenase 2 (CCD2) to produce crocetin dialdehyde, while the same reaction in *G. jasminoides* and *Buddleja davidii* is carried out by CCD4, which can catalyse the symmetric 7/8, 7′/8′ cleavage of zeaxanthin, β-carotene, and lycopene to produce crocetin dialdehyde [8,9,10,11]. The highly reactive crocetin dialdehyde then migrates to the endoplasmic reticulum (ER) and is dehydrogenated to crocetin by aldehyde dehydrogenase (ALDH) enzymes [8]. The final step of crocin biosynthesis involves the glycosylation of crocetin in the cytosol. This reaction is catalysed by members of the uridine diphosphate glycosyltransferase (UGT) superfamily, which usually mediates the glycosylation of secondary metabolites, xenobiotics, and hormones [12]. In addition, members of the ATP-binding cassette C (ABCC) transporter family, which are responsible for transporting crocins to the vacuolar lumen, play important roles in crocin accumulation [13]. However, our understanding of the regulation of crocin biosynthesis and accumulation in *G. jasminoides* remains limited. In *G. jasminoides*, only one CCD, one ALDH, and two UGTs have been functionally characterised in vitro [11,14]. However, several of these genes exhibit low expression levels in fruits, suggesting that they may not be the primary enzymes involved in crocin biosynthesis in vivo.

We usually refer to the process of converting genetic information from DNA to RNA as transcription [15]. The transcriptome refers to the general term for all the RNAs present in cells or tissues, which includes the well-known protein-coding RNAs (mRNAs) as well as a variety of other non-protein-coding RNAs (ncRNAs), such as long non-coding RNAs (lncRNAs), microRNAs (miRNAs), and circular RNAs (circRNAs) [16]. Transcriptomics provides a deeper understanding of the role of some functional components of the genome, such as transcriptome sequencing, which has widely revealed that the eukaryotic genome can transcribe a large number of ncRNAs that play an important role in regulating gene expression at the post-transcriptome level [17]. Currently, the most powerful tool for studying the transcriptome is transcriptome sequencing technology, with second-generation sequencing (or next-generation sequencing, NGS) represented by Illumina and third-generation sequencing (or next-next-generation sequencing, NNGS) represented by single-molecule real-time (SMRT) and Oxford Nanopore Technologies (ONT) being the most commonly used technologies [18]. NGS was developed on the basis of Sanger sequencing, which is favoured by researchers because of its high throughput, good accuracy, and low price [18]. However, NGS usually has short read lengths and cannot provide a large number of long transcripts and loses important information such as alternative splicing (AS), thus greatly limiting the in-depth utilisation of transcriptome data [18]. Compared with the short read length (50–500 bp) of NGS technology, the greatest advantage of NNGS in uncovering transcriptomics is that it can obtain full-length or near-full-length transcripts (more than 10 kb), which is important for AS identification, lncRNA analysis, novel gene discovery, and so on [18]. SMRT and ONT perform similarly in transcript identification and lncRNA prediction in transcriptome analysis, but SMRT excels in the identification of AS [19]. NNGS is being increasingly used in transcriptomic studies with its irreplaceable advantages and has been commonly exploited to generate full-length transcriptomes and identify key genes involved in major bioactive component biosynthesis in medical plant species, such as *Salvia miltiorrhiza*, *Astragalus membranaceus*, and *Pogostemon cablin* [20,21,22].

LncRNAs refer to non-coding RNAs with a length greater than 200 nt and a lack of a long open reading frame (ORF) and non-coding ability [23]. However, some lncRNAs could encode functional peptides [24]. Unlike miRNA, which has been thoroughly studied, lncRNA research started late. Initially, lncRNAs did not attract researchers’ attention and were regarded as transcriptional noise with a lack of function. A series of recent studies have shown that lncRNAs play important roles in many life activities and development processes [25]. LncRNAs can regulate the spatio-temporal expression of protein-coding genes via binding to transcription factors or participate in signal pathways as signal molecules. LncRNAs can indirectly regulate the expression of protein-coding genes by recruiting RNA-binding proteins such as transcription factors, chromosome modifications, and regulatory molecules that play important roles as decoy molecules. LncRNAs can guide riboprotein complexes to specific locations or recruit chromatin modification enzymes to target genes as a guide for RNA-binding proteins. LncRNAs can combine multiple proteins to form ribonucleoprotein complexes as scaffold molecules [26]. However, the mechanism of action of lncRNA is complex, and its regulatory mechanism has not been fully clarified. In recent years, many new lncRNAs have been discovered with new mechanisms of action. The explanation of the action mechanism of lncRNAs requires the joint efforts of a large number of researchers.

In previous work, we collected abundant wild and cultivated resources of *G. jasminoides* and found that there are variations in phenotypic traits, such as leaf type and fruit, among different cultivated lines [27]. In this study, the concentrations of crocin I were detected in different cultivated lines, and the line with the largest content of crocin was selected for transcriptome sequencing. We combined the concentrations of crocin I, SMRT, and Illumina sequencing to provide insight into the mechanisms of crocin biosynthesis and accumulation in the different fruit development stages of *G. gardenia*. We obtained a full-length reference transcriptome of *G. jasminoides* and added useful novel genes to the genome of the *G. jasminoides*. We identified putative genes and long non-coding RNAs involved in crocin biosynthesis and accumulation. In addition, we revealed the expression patterns of mRNA and lncRNAs in different fruit development stages of *G. gardenia*. Accordingly, this study not only provides a useful source for the further exploration of crocin biosynthesis but also provides a necessary reference for the targeted cultivation of *G. jasminoides*.

## 2. Results

### 2.1. Crocin Content Changes during Fruit Development

**Figure 1:**
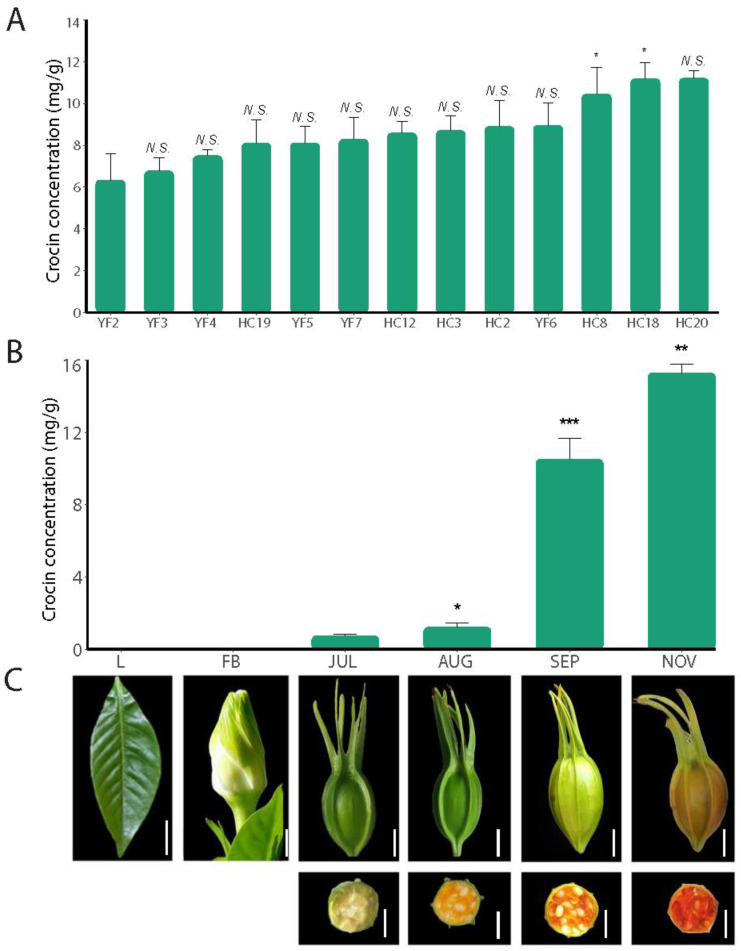
Morphology and crocin accumulation pattern of *G. jasminoides*. (**A**) The crocin I content determined in different cultivation lines of *G. jasminoides*. Starting with the second cultivation line, the significance at the top of each bar indicates whether there is a significant difference from the previous cultivation line. “N.S.” indicates no significant difference between the two cultivation lines, and “*” indicates a significant difference between the two cultivation lines (*p*-value less than 0.05). (**B**) Crocin I content in leaves, flower buds, and different fruit developmental stages. Mean values and standard deviations were obtained from three biological repeats. Starting with the third development stage, the significance at the top of each bar indicates whether there is a significant difference from the previous development stage. “*”, “**”, and “***” indicate the *p*-value is less than 0.05, 0.01, and 0.001, respectively. (**C**) Samples used in this study. Scale bar 1 cm.

Crocin I is the main bioactive component of gardenia fruits and is employed as an active ingredient in drugs. We collected a large number of germplasm resources of *G. jasminoides* and selected several excellent cultivation lines according to their yields and crocin I content. Among thirteen cultivation lines of *G. jasminoides*, the average concentrations of crocin I ranged from 6.34 mg/g to 11.20 mg/g fresh weight (FW) (Figure 1A). The HC18 and HC20 lines had higher crocin I content than the other lines. In addition, the HC18 line is one of the main cultivation types of *G. jasminoides* in Jiangxi Province because of its large fruits and high yield. Therefore, the HC18 line was selected for transcriptome sequencing. HPLC analysis of different organs and tissues showed that crocin I was specifically located in the fruit and that the content of crocin I gradually increased during fruit maturation from JUL to NOV (Figure 1B), suggesting that crocin I biosynthesis may be carried out in the fruit. Additionally, with the development of HC18 fruit, the peel changes from green to reddish yellow, and the pulp changes from beige to its characteristic reddish-orange colour, showing a change in colour across the gradient of fruit maturation (Figure 1C). It is worth noting that from AUG to SEP, the content of crocin I increased rapidly from 1.22 mg/g to 10.52 mg/g and reached a peak at the beginning of November, indicating that crocin biosynthesis and accumulation mainly occurred from SEP to NOV.

### 2.2. Pacbio Long-Read Sequencing Data Analysis

**Figure 2:**
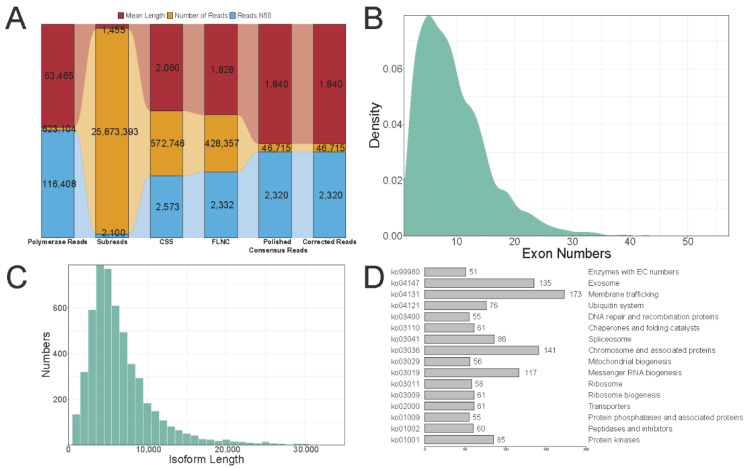
SMRT sequencing data statistics. (**A**) The upstream data statistics of SMRT sequencing. (**B**) The exon numbers distribution of novel isoforms. (**C**) The length distribution of novel isoforms. (**D**) The KEGG annotation of novel isoforms.

High-quality RNAs from leaves, flowers, and four different developmental stages of fruits (JUL, AUG, SEP, and NOV) were combined equally to construct the isoform-sequencing library. After sequencing on the PacBio Sequel II platform, we yielded 623,104 polymerase reads (N50 = 116,408 bp) with 39.55 billion nucleotides and an average length of 63,465. A total of 25,873,393 subreads (N50 = 2100 bp) with 37.64 billion nucleotides and an average length of 1455 after removing the adapter sequence and reads shorter than 50 bp. A total of 572,746 circular consensus sequences (CCS, N50 = 2573 bp) with an average length of 2060 were obtained by filtering less than one supported subread. Using SMRTlink software, we detected 428,357 full-length non-chimaera reads (FLNC, N50 = 2332 bp, reads with 5′ primer, 3′ primer, and poly-A sequences), 127,383 non-full-length non-chimaera reads (NFL), 445,363 full-length reads (FL), and 16,259 full-length chimaera reads (FLC). A total of 46,715 polished consensus reads (N50 = 2320 bp) were obtained by clustering the FLNC reads and polishing them with arrow software. Finally, we obtained 46,715 corrected isoform sequences (N50 = 2320 bp) with an average length of 1840 by using LoRDEC software with Illumina reads as criteria (Figure 2A). The corrected isoform sequences occur frequently in the 1000 to 4000 bp range, with only a few sequences exceeding 6000 bp (Figure S1).

### 2.3. Identification and Annotation of Novel Transcripts

A total of 46,085 (98.65%) corrected isoform sequences mapped to the genome of *G. jasminoides*, with 23,011 and 23,074 corrected isoforms could map to the plus strand and minus strand, respectively. Based on the mapped results, we identified 147 novel transcripts of 143 novel genes and 5083 novel transcripts of 4500 known genes. A total of 5230 novel isoforms occur frequently in the 1000 to 10,000 bp range, with only a few sequences exceeding 20,000 bp (Figure 2C). Most of the 5230 novel isoforms have fewer than 20 exons, and only a small fraction have more than 30 exons (Figure 2B). A total of 1447 novel isoforms could be annotated in the 135 KEGG pathways, of which 16 pathways contained more than 50 novel isoforms. The top three KEGG pathways were “membrane trafficking”, “chromosome and associated proteins”, and “exosome” (Figure 2D, Table S1). A total of 214 novel isoforms could be annotated as transcription factors (TFs), and the top five TFs were “C3H”, “AUX/IAA”, “SNF2”, “MYB-related”, and “NAC” (Table S2). The remaining 630 unmapped corrected isoforms were compared to the NR, NT, Pfam, KOG/COG, KEGG, and GO databases to perform functional annotation. Of the six databases, the NR and KOG/COG databases annotated the most isoform transcripts (525), followed by PFAM (463), GO (282), KEGG (213), and NT (178). A total of 113 unmapped corrected isoforms (17.94%) are annotated in all six databases, and 528 were annotated in at least one database (Figure S2). The top three terms in biological processes were “cellular process”, “metabolic process”, and “response to stimulus”; the top two terms in cellular components were “cellular anatomical entity” and “protein−containing complex”; and the top three terms in molecular function were “binding”, “catalytic activity”, and “structural molecule activity” (Figure S3). A total of 213 unmapped corrected isoforms were distributed into 153 KEGG terms. The top three terms were “metabolic pathways”, “ribosome”, and “biosynthesis of secondary metabolites”.

### 2.4. LncRNA and AS

**Figure 3:**
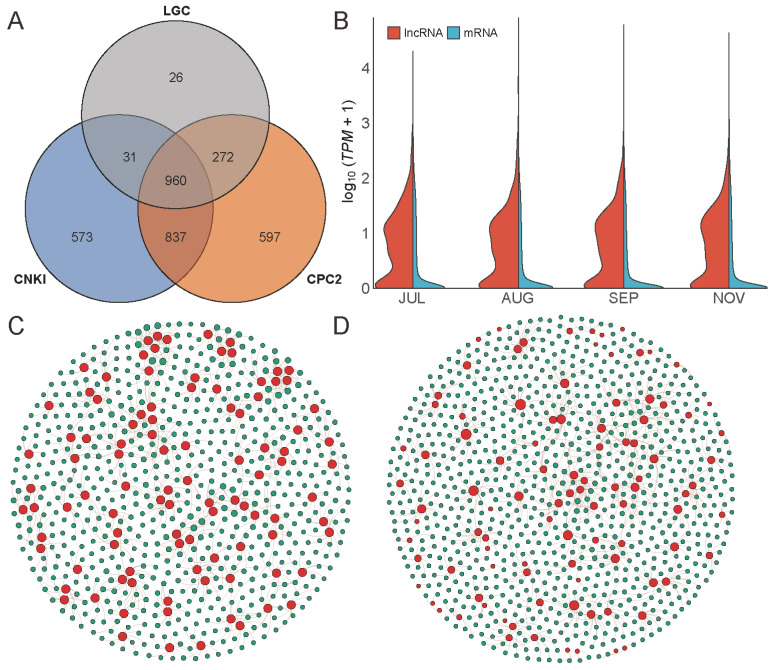
The lncRNAs in *G. jasminoides*. (**A**) Venn diagram of lncRNA identification results by LGC, CNKI, and CPC2. By combining three programs, a total of 960 lncRNAs were identified. (**B**) The expression profile of lncRNAs and mRNAs. (**C**,**D**) The *cis* and *trans* target genes of 960 lncRNAs, with the red point representing lncRNAs and the green point representing target genes.

A total of 960 lncRNAs were identified by combining CPC2, CNKI, and LGC (Figure 3A). Based on the genome position of lncRNAs, they could be divided into four subclasses: lincRNA (86), antisense lncRNA (9), sense intronic lncRNA (4), and sense overlapping (861) (Figure S4). The length of most mRNAs ranged from 0 to 10,000 bp, while almost all lncRNAs were shorter than 5000 bp (Figure S5). The exon numbers of lncRNAs were less than 10, while most mRNAs were less than 20 (Figure S6). It is, therefore, that the length and exon numbers between lncRNAs and mRNAs are larger differences. The expression pattern of lncRNAs in different developmental stages of fruits was strikingly similar, and almost all lncRNA *TPM* values were less than 10; the same situation occurred in the expression pattern of mRNAs (Figure 3B). In addition, we found that 960 lncRNAs can target 13,484 genes in *cis* and 12,711 genes in *trans*. LncRNAs and their target genes form a very complex regulatory network. Most lncRNAs could target more than one gene. For example, the lncRNA MSTRG_6272_1 could target 41 different genes in *cis* and one gene in *trans*. One gene could be targeted by one or more different lncRNAs. For example, the Gj2A2T70 gene could be targeted by seven lncRNAs in *cis* and 13 lncRNAs in *trans* (Figure 3C,D, Tables S3 and S4).

**Figure 4:**
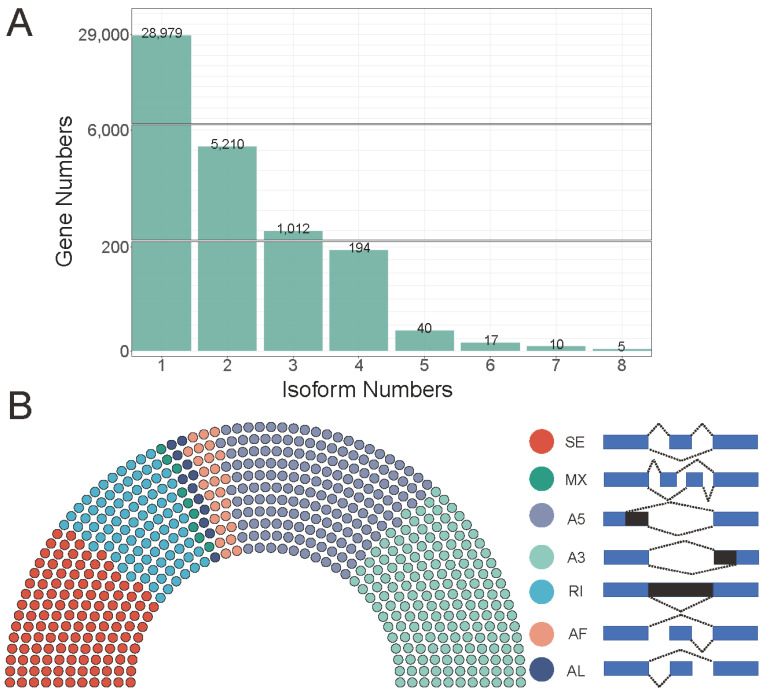
The transcript profile of *G. jasminoides*. (**A**) Distribution of genes that produce one or more splice isoforms in *G. jasminoides*. The number of genes is shown at the top of each bar. (**B**) The total number of AS events in *G. jasminoides* represented by one point for every ten AS events.

Stringtie and gffcompare were used to reconstruct the transcript of *G. jasminoides*, and we discovered that 28,979, 5210, 1012, 194, 40, 17, 10, and 5 genes generated one, two, three, four, five, six, and eight isoform numbers, respectively (Figure 4A). Then, SUPPA software was used to obtain the seven AS types (skipping exon, SE; mutually exclusive exons, MX; alternative 5′ splice-site, A5; alternative 3′ splice-site, A3; retained intron, RI; alternative first exon, AF; alternative last exon, AL). A total of 6052 AS events were identified from 3823 genes, of which A3 was the most abundant, accounting for 28.40% of all AS events. The following are A5 (1663, accounting for 27.47%), SE (1297, accounting for 21.43%), RI (902, accounting for 14.90%), AF (263, accounting for 4.35%), AL (119, accounting for 1.90%), and MX (89, accounting for 1.47%). KEGG enrichment analysis showed that the AS genes were significantly enriched in “pentose phosphate pathway”, “fatty acid biosynthesis”, “carbon fixation in photosynthetic organisms”, “alanine, aspartate and glutamate metabolism”, “aminoacyl−tRNA biosynthesis”, “propanoate metabolism”, and so on (Figure S7, Table S5).

### 2.5. Gene Expression Profile during Fruit Development

**Figure 5:**
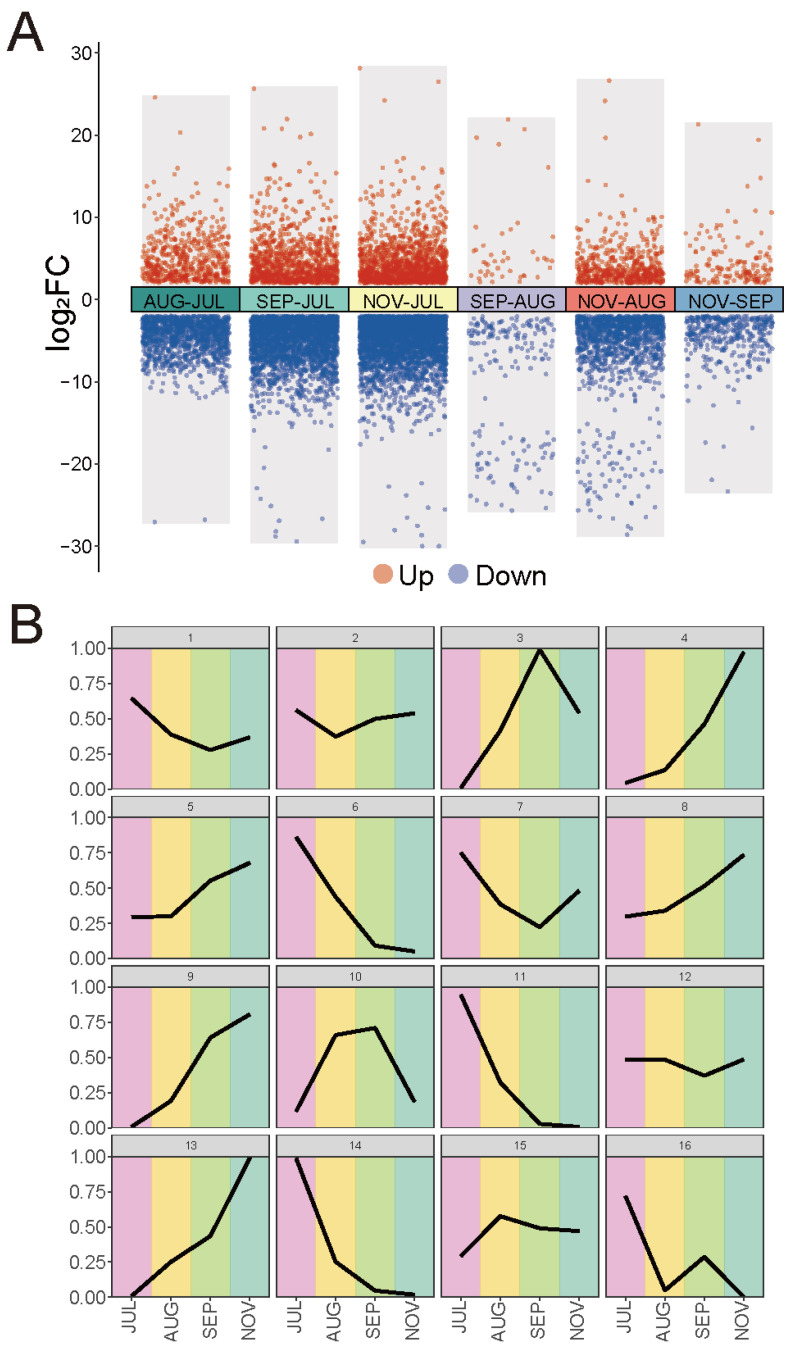
Gene expression profile during fruit development in *G. jasminoides*. (**A**) The DEGs at various stages of fruit development, with the red point representing up genes and the blue point representing down genes. (**B**) Mclust-generated maximum-normalised expression values for genes in the top 10% of expression variance in each of the 16 clusters.

To better understand the gene expression dynamics during fruit development, we evaluated the gene expression profile in three ways. First, a total of 6216 differentially expressed genes (DEGs) were identified during fruit development, with an FDR cut-off of less than 0.05 and a |log_2_(FoldChange)| ≥ 2 (Figure 5A and Figure S8). According to the DEG analysis results, NOV and JUL had the most DEGs (4550). The following are SEP and JUL (3564), AUG and JUL (1769), NOV and AUG (1755), NOV and SEP (520), and SEP and AUG (217). The DEGs between JUL and the other three stages of the fruit became progressively more numerous, suggesting that these genes may play an important role in fruit development (Figure 5A and Figure S8). The KEGG pathway enrichment results showed that the top three enriched terms in AUG vs. JUL were “stilbenoid, diarylheptanoid, and gingerol biosynthesis”, “pentose and glucuronate interconversions”, and “flavonoid biosynthesis”. The top three enriched terms in SEP vs. JUL were “brassinosteroid biosynthesis”, “pentose and glucuronate interconversions”, and “zeatin biosynthesis”. The top three enriched terms in NOV vs. JUL were “zeatin biosynthesis”, “fatty acid elongation”, and “diterpenoid biosynthesis” (Figure S9, Table S6). The biosynthesis of crocin includes the upstream MEP and MVA pathways, the midstream carotenoid biosynthesis pathway, and the downstream crocin biosynthesis pathway. Some of the DEGs were enriched in terpenoid biosynthesis pathways, indicating that they may be involved in the biosynthesis of crocin. We also found some AS genes were DEGs. Among the 3823 genes that have at least one AS event, 463 were significantly differently expressed in at least one compared group (Table S7). A total of 96 AS genes were significantly differently expressed in AUG compared to JUL, including 38 up-regulated genes and 58 down-regulated genes. A total of 238 AS genes were significantly differently expressed in SEP compared to JUL, including 120 up-regulated genes and 118 down-regulated genes. A total of 347 AS genes were significantly differently expressed in NOV compared to JUL, including 171 up-regulated genes and 176 down-regulated genes. A total of nine AS genes were significantly differently expressed in SEP compared to AUG, including five up-regulated genes and four down-regulated genes. A total of 136 AS genes were significantly differently expressed in NOV compared to AUG, including 67 up-regulated genes and 69 down-regulated genes. A total of 34 AS genes were significantly differently expressed in NOV compared to SEP, including 6 up-regulated genes and 28 down-regulated genes (Table S7).

Second, we used the τ score to measure the specific expression potential of four developmental stages of all genes. A total of 3600 genes were considered to have a high potential for stage-specific expression in fruit development with a τ score ≥ 0.95, of which 2356 genes were only expressed in one stage of fruit development, indicating that they have high expression specificity (Table S8). According to the heatmap cluster results, we found that most of the high stage-specific expression in fruit development tended to be highly expressed in JUL (Figure S10). The KEGG enrichment of 3600 genes indicated that they were enriched in “phenylpropanoid biosynthesis”, “sesquiterpenoid and triterpenoid biosynthesis”, “stilbenoid, diarylheptanoid and gingerol biosynthesis”, “starch and sucrose metabolism”, “flavonoid biosynthesis”, “glucosinolate biosynthesis”, and “diterpenoid biosynthesis” (Figure S11). Third, the R package mclust was used to perform the time series analysis and based on the results, the optimal cluster number was 16 (Figure 5B and Figure S12). These 16 distinct covariance clusters were organised into the following groups: (A) expression rose with fruit development; (B) expression decreased with fruit development; (C) expression increased and then decreased with fruit development; and (D) expression decreased and then increased with fruit development. We found some AS genes tend to have tissue-specific expression. Among the 3823 AS genes, 76 have tissue-specific expression with a τ score larger than 0.95. A total of 39 AS genes were only expressed in one development stage of fruit. MSTRG_10155, MSTRG_10443, and MSTRG_2153, for example, were only expressed in JUL; MSTRG_1621, MSTRG_1798, and MSTRG_1810, only in AUG; MSTRG_192, MSTRG_4204, and MSTRG_6999, only in SEP; and MSTRG_10162, MSTRG_1146, and MSTRG_1482, only in NOV. We found the expression levels of these AS genes with τ score equal to one were extremely low. The remaining AS genes with τ score larger than 0.95 were expressed in at least two development stages of fruits, but expression levels in different development stages of fruits showed quite drastic variation. For example, MSTRG_2198 with τ score larger than 0.99 was expressed in AUG (*TPM* = 0.05) and NOV (*TPM* = 12.89). The fold change between the two exceeded 250; MSTRG_2481 with τ score larger than 0.98 was expressed in JUL (*TPM* = 39.27), AUG (*TPM* = 1.69), and SEP (*TPM* = 0.11). The fold change between JUL and SEP exceeded 350 (Table S9).

### 2.6. Key Genes and lncRNAs Involved in Crocin Biosynthesis

**Figure 6:**
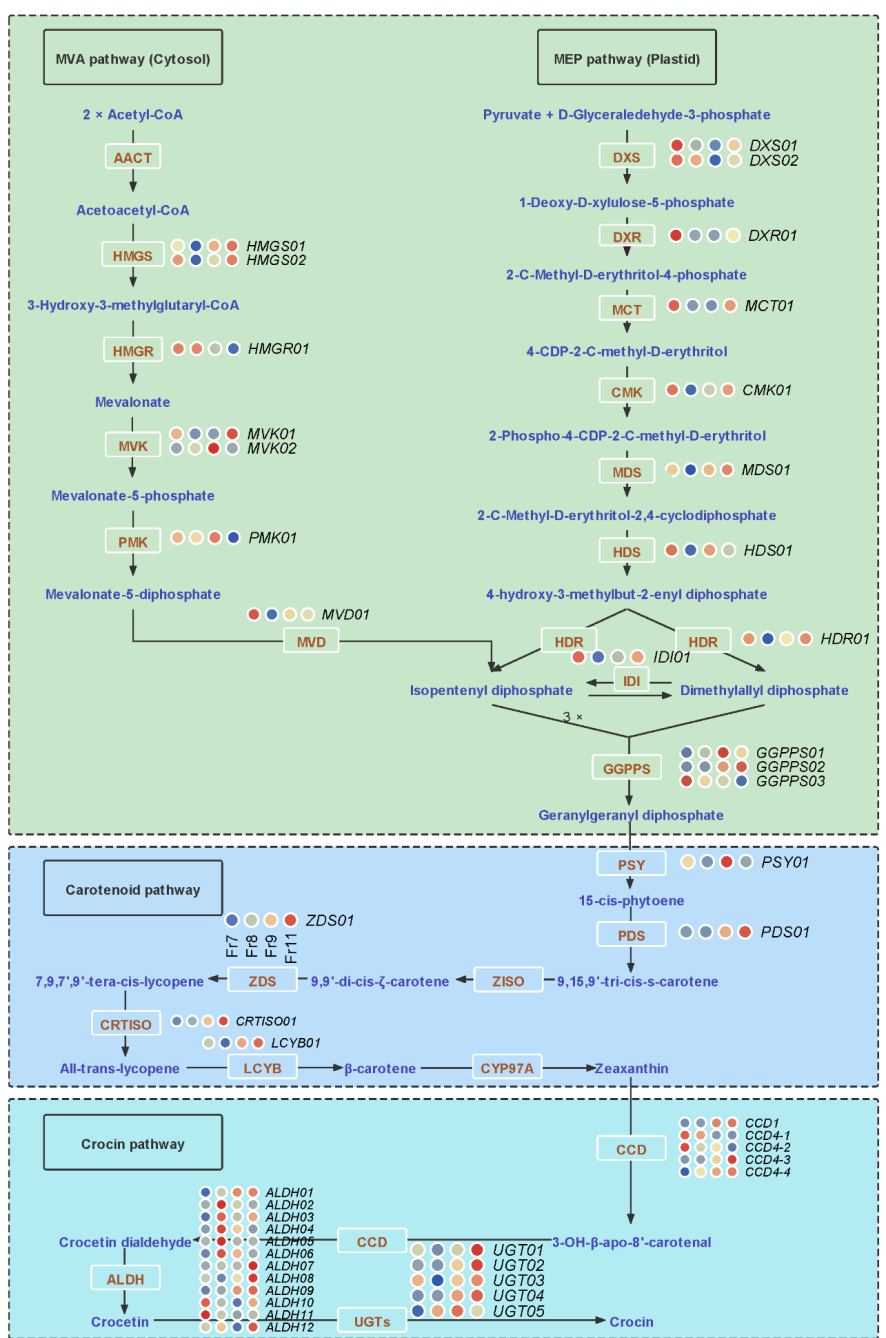
Regulatory and synthetic networks of crocin biosynthesis in *G. jasminoides*. Tissue-specific relative expression profiles (red–blue scale) of genes implicated in crocin biosynthesis (heatmap). JUL (green peel and white pulp); AUG (green peel and light-yellow pulp); SEP (light-yellow peel and orange pulp); NOV (red peel and orange pulp).

**Figure 7:**
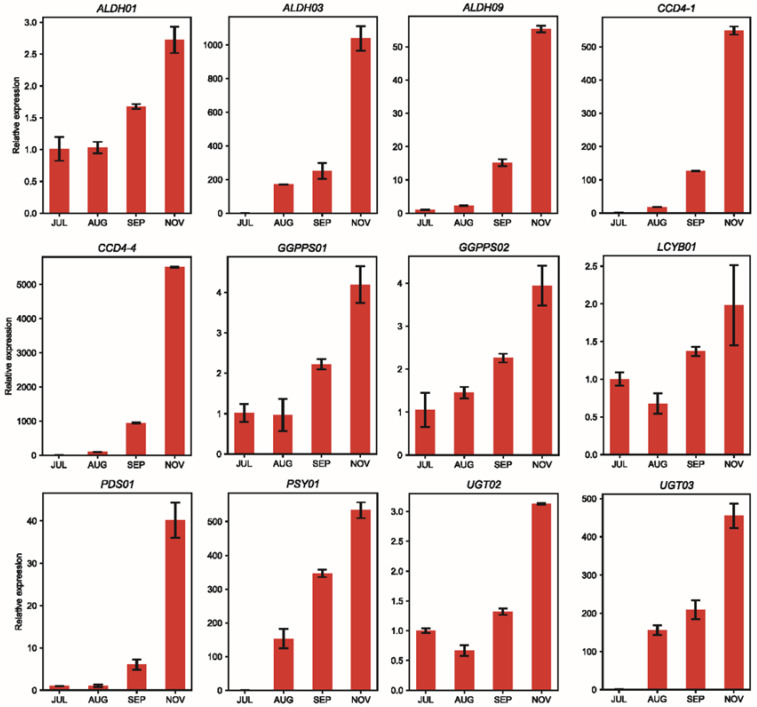
Tissue and temporal expression patterns of candidate genes involved in crocin biosynthesis determined by qRT-PCR. The relative expression levels were calculated according to the 2^−ΔΔCT^ method using *Gj18S* as an internal reference gene. Error bars represent standard deviations.

Crocin biosynthesis includes three parts: the upstream MEP and MVA pathways, the midstream carotenoid biosynthesis pathway, and the downstream crocin biosynthesis pathway [28]. To gain insight into crocin biosynthesis at the gene and enzyme levels, a heatmap based on *TPM* during different fruit developmental stages was analysed to illustrate the expression profiles of candidate genes involved in crocin biosynthesis and accumulation. A total of 46 genes that may be involved in crocin biosynthesis were identified in this study, including 19 genes in the upstream pathway (two *HMGS* genes, one *HMGR* gene, two *MVK* genes, one *PMK* gene, one *MVD* gene, two *DXS* genes, one *DXR* gene, one *MCT* gene, one *CMK* gene, one *MDS* gene, one *HDS* gene, one *HDR* gene, one *IDI* gene, and three *GGPPS* genes), five genes in the midstream pathway (one *PSY* gene, one *PDS* gene, one *ZDS* gene, one *CRTISO*, and one *LCYB* gene), and 22 genes in the downstream pathway (five *CCD* genes, 12 *ALDH* genes, and five *UGT* genes). Most of the genes involved in crocin biosynthesis (36 of 46) were highly expressed in JUL and NOV, and ten of 46 genes were DEGs. (Figure 6, Table S10). For example, the *TPM* values of *ALDH07* in JUL, AUG, SEP, and NOV were 153.31, 169.11, 164.93, and 452.51, respectively. The expression of *ALDH07* in JUL, AUG, and SEP was almost constant. However, the expression in NOV was almost three times as high as before, which implied that the high expression of *ALDH07* in NOV may be key to crocin biosynthesis (Figure S13). Additionally, some AS genes were involved in crocin biosynthesis, including nine A5 type genes (*ALDH03*, *ALDH06*, *ALDH11*, *CCD4-1*, *DXS02*, *HDS01*, *MVD01*, *PDS01*, and *PMK01*); three A3 type genes (*ALDH10*, *ALDH11*, and *DXR01*), three RI type genes (*ALDH11*, *DXS02*, and *ZDS01*); two SE type genes (*CCD4-4* and *MCT01*); and one AL type gene (*ALDH01)*. Furthermore, 12 candidate genes responsible for crocin biosynthesis were verified by qRT-PCR analysis, indicating the reliability of the RNA-seq results (Figure 7).

**Figure 8:**
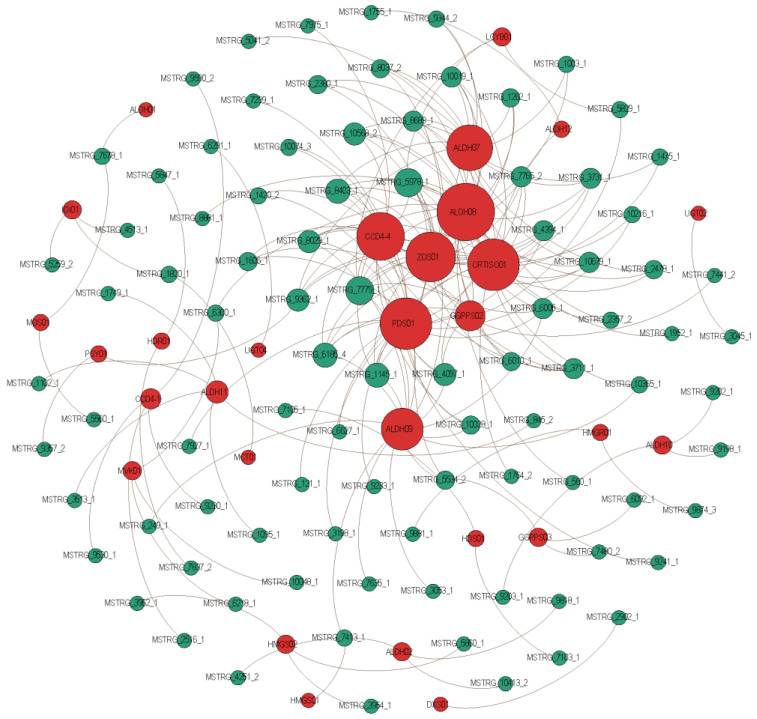
The lncRNAs involved in the crocin I biosynthesis pathway. The red and green points represent lncRNAs and target genes, respectively. Grey lines indicate that lncRNAs may have the potential ability to regulate the connected protein genes.

As a member of a class of non-coding RNAs that are receiving increasing attention from researchers, lncRNAs can regulate genes that are close together in *cis* or far away in *trans*, thus participating in the hierarchical network of gene expression regulation. A total of 91 lncRNAs that may be involved in crocin biosynthesis were identified in this study (Figure 8). They could target 29 genes that are involved in crocin biosynthesis and form 189 regulation pairs, of which 21 lncRNAs could target 13 genes in *cis*, while 73 lncRNAs could target 23 genes in *trans*. We found that one gene could be targeted by more than one lncRNA. For example, *ALDH07* could be targeted by 17 lncRNAs, of which one was in *cis*, and 16 were in *trans*; *ALDH08* could be targeted by 23 lncRNAs, of which one was in *cis*, and 22 were in *trans*; and *CRTISO01* could be targeted by 20 lncRNAs in *trans*. One lncRNA could also target more than one gene. For example, lncRNA MSTRG_9362_1 could target four genes in *trans* (*ALDH07*, *ALDH08*, *CRTISO01*, and *ZDS01*) and one gene in *cis* (*PSY01*); lncRNA MSTRG_5534_2 could target one gene in *trans* (*GGPPS03*) and two genes in *cis* (*ALDH08* and *ALDH09*); lncRNA MSTRG_5978_1 could target eight genes in *trans* (*ALDH08*, *ALDH09*, *CCD4-4*, *CRTISO01*, *GGPPS02*, *LCYB01*, *PDS01*, and *ZDS01*). The target genes of those lncRNAs are all key genes involved in crocin biosynthesis, which implies that those lncRNAs may be key to crocin biosynthesis (Figure 8, Table S11).

Thirty-one out of 91 lncRNAs take part upstream of crocin biosynthesis (Figure 8). The *HMGR*, *HMGS*, *MVK*, *DXS*, *MCT*, *MDS*, *HDS*, *HDR*, *IDI*, and *GGPPS* genes could be targeted by 2, 4, 3, 1, 1, 2, 2, 2, 3, and 12 lncRNAs, respectively. The *GGPPS* gene was the target gene for most of the 31 lncRNAs, which was a catalyst for three isopentenyl diphosphates and one dimethylallyl diphosphate to form geranylgeranyl diphosphate, which was the last product of the upstream. These lncRNAs may be key to crocin biosynthesis. Thirty-eight out of ninety-one lncRNAs take part in the midstream of crocin biosynthesis. The *PSY*, *PDS*, *ZDS*, *CRTISO*, and *LCYB* genes could be targeted by 2, 20, 19, 20, and 3 lncRNAs, respectively. The *PDS*, *ZDS*, and *CRTISO* genes were the target genes for most of the 38 lncRNAs. The *PDS* gene could catalyse 15-cis-phytoene to form 9,15,9’-tri-cis-s-carotene, and the *ZDS* gene could catalyse 9,9’-di-cis-ζ-carotene to form 7,9,7’,9’-tera-cis-lycopene, and then to form all-trans-lycopene via the *CRTISO* gene. A complex regulatory network comprised of 38 lncRNAs and five protein-coding genes was formed. It is implied that they may play a key role in crocin biosynthesis. Sixty-three out of ninety-one lncRNAs take part downstream of crocin biosynthesis. The *CCD*, *ALDH*, and *UGT* genes could be targeted by 22, 49, and 2 lncRNAs, respectively. The *ALDH* gene was the target gene for most of the 63 lncRNAs. It could catalyse crocetin dialdehyde to form crocetin. Interestingly, seven lncRNAs (MSTRG_1145_1, MSTRG_1606_1, MSTRG_3711_1, MSTRG_4097_1, MSTRG_5978_1, MSTRG_6185_4, and MSTRG_7779_1) could take part in all three layers of crocin biosynthesis by targeting different protein-coding genes.

Based on the *TPM* values, we found that most of the lncRNAs involved in crocin I biosynthesis tended to be lowly expressed in JUL, AUG, and SEP and highly expressed in NOV (Figure S14). More than half of the 91 lncRNAs involved in crocin I biosynthesis tended to be highly expressed in NOV. For example, the *TPM* values of MSTRG_1003_1 (targeting *ALDH07* and *ALDH08* genes in *trans*) in JUL, AUG, SEP, and NOV were 7.27, 7.84, 7.90, and 34.13, respectively. It was almost consistently expressed in JUL, AUG, and SEP, but nearly five-fold higher in NOV than in the first three development stages. The *TPM* values of MSTRG_1262_1 (targeting *ZDS01*, *ALDH07*, and *ALDH08* genes in *trans*) in JUL, AUG, SEP, and NOV were 0.17, 0.20, 0.16, and 1.37, respectively. It was barely expressed at the first three development stages, but highly expressed at the NOV. The same lncRNAs also have MSTRG_1420_2 (targeting *CCD4-4* and *PDS01* genes in *trans*), MSTRG_2478_1 (targeting *ALDH07*, *ALDH08*, and *CRTISO01* genes in *trans*), and MSTRG_9344_2 (targeting *ALDH07* and *ALDH08* genes in *trans*). In addition, some lncRNA expression patterns are diametrically opposed to the above-described lncRNAs. For example, MSTRG_7480_2 (targeting the *GGPPS03* gene in *trans*), had *TPM* values of 266.09, 119.99, 0.03, and 0 at four development stages, respectively. Similar lncRNAs also have MSTRG_9590_2 (targeting the *CCD4-1* gene in *trans*), and MSTRG_9230_1 (targeting the *CCD4-1* gene in *trans*). The above results suggest that the lncRNAs involved in crocin I biosynthesis are regulated in a complex pattern. We also found that some lncRNAs were significantly differentially expressed at different developmental stages. For example, MSTRG_6092_1 (targeting the *GGPPS03* gene in *trans*) was differentially expressed in AUG and JUL; MSTRG_1606_1 (targeting *GGPPS02*, *CCD4-4*, and *PDS01* genes in *trans*), MSTRG_845_2 (targeting the *ALDH11* gene in *trans*), and MSTRG_1749_1 (targeting the *ALDH11* gene in *trans*) were differentially expressed in SEP and JUL; MSTRG_1262_1 (targeting *ZDS01*, *ALDH07*, and *ALDH08* genes in *trans*), MSTRG_1420_2 (targeting *CCD4-4* and *PDS01* genes in *trans*), and MSTRG_1475_1 (targeting *ALDH07* and *CRTISO01* genes in *trans*) were differentially expressed in NOV and JUL (Table S12).

## 3. Discussion

Plants produce various secondary metabolites during growth and development, and these metabolites are mainly in three major groups: phenolics (18%), terpenoids (54%), and alkaloids (27%) [29]. Phenolic compounds play an important role in plant-environment interactions, such as flavonoids that have significant resistance to plant pathogens and anthocyanins that effectively eliminate reactive oxygen species (ROS) to mitigate UV-induced damage [30]. Alkaloids are nitrogen-containing bioactive substances that play an important role in plant resistance to biotic and abiotic stresses. For example, when tobacco (*Nicotiana tabacum*) leaves are traumatised by diseases, herbivores, and insects, root cells synthesise large amounts of nicotine, which is transported to the leaf vacuole by nicotine transporter proteins to resist biotic and abiotic stresses [31]. Terpenoids are the most diverse group of plant secondary metabolites and have important physiological and ecological functions. For example, the essential oil of *Cinnamomum camphora* is rich in monoterpenes and sesquiterpenes that have various biological activities, such as antibacterial, antioxidant, and anti-inflammatory activities, and has been widely used in the light industry, spice industry, beauty industry, and manufacturing industry [32]. Because of the long read lengths involved, SMRT sequencing based on the PacBio platform, representing NNGS technology, can provide high-quality and more complete transcripts in genomic and transcriptomic investigations [33,34]. Full-length transcripts provide more information and address the problems of transcript redundancy and incomplete assembly that occur with NGS technology. An increasing number of studies are based on transcriptome sequencing to study the process of secondary metabolite accumulation in plants. For example, Kuang et al. used NNGS technology to study the biosynthesis process of taxol in *Taxus cuspidata* and found that nine cytochrome P450s (CYP450s) and seven BAHD acyltransferases might be associated with taxol biosynthesis [35]. Chen et al. used NGS technology to study terpenoid biosynthesis in the camphor tree and found that miR4995, miR5021, and miR6300 might be involved in terpenoid biosynthesis [32].

Full-length transcriptome sequencing is widely used for the identification of novel genes, lncRNAs, and AS events because of its feature of not having to interrupt mRNA. In this study, 147 novel transcripts of 143 novel genes and 5083 novel transcripts of 4500 known genes were obtained based on full-length transcriptome sequencing. In addition, we identified 960 lncRNAs and 6052 AS events derived from 3823 genes. This information greatly complements the genome of *G. jasminoides* and provides new insight into the biosynthesis of its active substances. A large number of studies have been conducted based on the full-length transcriptome. For example, Feng et al. identified 25,246 genes, 1468 lncRNAs, and 31,448 AS events in *Gossypium australe*; and found that RI was the most frequent AS event (accounting for 68.85%) [36]. Wang et al. identified 21,448 novel genes, 320 lncRNAs, and 31,448 AS events in *Ricinus communis* [37]. Tan et al. identified 42,683 novel isoforms, 407 lncRNAs, and 453,270 AS events in *Brassica rapa* L. ssp. *pekinensis* [38].

There were reports that studied the accumulation mechanism of crocin I in *G. jasminoides* based on NGS technology, but there has been no report on crocin biosynthesis based on NNGS technology [39,40]. Based on SMRT and Illumina sequencing technologies, A total of 46 protein-coding genes involved in crocin I biosynthesis were identified in this study, with 19, 5, and 22 genes involved in the upstream, midstream, and downstream pathways, respectively. In addition, more than half of the genes were highly expressed in NOV, which is consistent with the results of the metabolomics; a total of 10 genes were significantly differentially expressed in different developmental stages of fruit, such as *CCD4-4*, *HMGS02*, *ALDH01*, *ALDH09*, and *GGPPS02* genes. The *CCD4-4* was 15 times more expressed in NOV than in JUL and was the most variable gene. Pan et al. sequenced fruits of different colours of *G. jasminoides* and identified 32 genes involved in crocin I biosynthesis. A total of 14, 4, and 14 genes in the upstream, midstream, and downstream pathways, respectively. *FPPS1*, *DXS1*, and *DXS3* were highly expressed in FP (the peel of red fruit). A total of 14 key DEGs (three *CCD*, two *NCED*, five *ALDH*, and four *UGT* genes) were detected as differently expressed [39]. Ji et al. sequenced the leaves, green fruits, and red fruits of *G. jasminoides* and identified 36 genes involved in crocin I biosynthesis. There were a total of 13 and 23 genes in the midstream and downstream pathways, respectively. Most of the genes were highly expressed in red fruits, among which 23 genes were specifically expressed in the fruits [40].

In addition to protein-coding genes, some non-coding regulators may also play an important role in crocin I biosynthesis. Some studies reported protein-coding genes involved in crocin I biosynthesis, but few studies reported the long non-coding RNAs involved in crocin I biosynthesis. In this study, we also identified 91 lncRNAs that may be potentially involved in crocin I biosynthesis. A total of 31, 38, and 63 lncRNAs were potentially involved in the upstream, midstream, and downstream of crocin biosynthesis, respectively. LncRNAs and other non-coding RNAs, such as microRNAs (miRNAs) and circular RNAs (circRNAs), were initially considered transcriptional “noise” without function [41]. In recent years, there has been increasing evidence showing that lncRNAs, although they do not encode proteins, can function in less familiar ways and are thus involved in various stages of plant growth and development [42]. Numerous studies have also revealed the role of lncRNAs in plant secondary metabolites. Lignin is one of the major components of wood and one of the important renewable energy sources on Earth [43,44]. Quan et al. found that some lncRNAs may regulate some protein-coding genes in the lignin biosynthesis pathway in a cis or trans manner, providing new insights into wood formation [45]. Kuntala et al. discovered 113 and 29 lncRNAs involved in the terpenoids and flavonoids metabolic pathway of *Citrus limon*, respectively [46]. These studies imply that lncRNAs may have a wide range of functions.

## 4. Materials and Methods

### 4.1. Sample Preparation

In October 2018, the fruits of thirteen *G. jasminoides* cultivation lines (3 clones per line) were collected from six-year-old trees that were planted in an experimental field in Gongqingcheng (29.19° N, 115.78° E), and the content of crocin was determined. The line with the higher content of crocin and a high yield was selected for transcriptome sequencing. In 2019, leaf, flower bud, and fruit samples from four developmental stages were collected from the selected trees with three biological replicates. The four sampling stages were defined as JUL (green peel and white pulp), AUG (green peel and light-yellow pulp), SEP (light-yellow peel and orange pulp) and NOV (red peel and orange pulp) according to harvesting time. All the samples were immediately frozen in liquid nitrogen and stored at −80 °C.

### 4.2. Crocin Measurement

The crocin contents of the fruit samples from four developmental stages were analysed by HPLC. A crocin I standard was purchased from Shanghai Yuanye Biological Technology Co., Ltd. (Shanghai, China) (B21337 and purity ≥ 98%) and was solvated in methanol at a level of 28 µg/mL. The dried powder of each sample (100 mg fresh weight) was dissolved in 25 mL of methanol for 20 min by ultrasonic-assisted extraction (30 °C, 16 kHz). The mixed solutions were then filtered through a 0.22 µm syringe filter. Each sample’s 20 µL extract solution was quantified using HPLC (LC-20AT, SHIMADZU), with the column temperature set to 30 °C and the flow rate set to 1.0 mL/min. A Waters C18 column (4.6 × 250 mm, 5 μm) and a mobile phase consisting of Solvent A (acetonitrile) and Solvent B (1% caproic acid/water (*v*/*v*)) were used for this analysis. The mobile phase gradient was as follows: 0–15 min, 20–60% A; 15–20 min, 90% A; 20–25 min, 20% A. Finally, the absorption of the aqueous phase was measured at 440 nm (Crocin I).

### 4.3. PacBio Library Construction and Sequencing

Total RNA was extracted from each sample with the RNeasy Plus Mini Kit (Qiagen, Germany). The integrity of the total RNA was evaluated using an Agilent 2100 Bioanalyser (Agilent Technology, Santa Clara, CA, USA). The purified RNAs from 3 different tissues and 4 different stages of fruit were mixed equally and used for PacBio library construction and sequencing. cDNA was synthesised using the Clontech SMARTer PCR cDNA Synthesis Kit. PCR fragments of 0.5–6 kb were retained using the BluePippin™ Size Selection System. The ends of the cDNA were repaired, ligated to adapters, and sequenced on the PacBio Sequel platform.

### 4.4. Transcriptome Data Analysis

SMRTlink (v7.0) was employed to process the SMRT raw sequence data. First, subreads were filtered to obtain a circular consensus sequence (CCS). According to whether the sequence included 5′ primers, 3′ primers, and polyA tails, CSSs were further divided into full-length nonchimeric (FLNC) sequences and non-full-length sequences. Subsequently, the FLNC sequences were clustered to generate polished consensus isoforms using arrow software and further corrected using filtered Illumina RNA-Seq reads with LoRDEC [47].

LncRNAs are a class of RNA molecules with transcripts longer than 200 nt that do not encode proteins. In this study, lncRNA identification was performed based on the feature that lncRNAs do not encode proteins. Firstly, corrected isoforms of SMRT reads were aligned to the genome of *G. jasminoides* using gmap software to obtain the sam files [48]; then, Stringtie and gffcompare software were used to reconstruct the transcript based on the sam files obtained in the previous step [49,50]; a shell script was used to filter candidate lncRNAs based on class_code information (class_code of “u”, “x”, “i”, “j”, or “o”). Then the sequences with a transcript length of less than 200 nt were filtered out. Finally, the transcripts that passed the screening were analysed for coding potential using CPC, CNCI, and LGC with default parameters [51,52,53]. An in-house shell script was used to obtain the lncRNAs that were considered not to have coding potential by all three programs. Unlike miRNAs, which can identify target genes based on whether they can complementarily pair with target sequences, lncRNAs have a complex and diverse mechanism of action and can regulate target genes in *cis* or *trans*. Genes that are close to the location of lncRNAs are regulated in *cis*, while genes that are far from the location of lncRNAs are regulated in *trans*; thus, the expression level of the target gene is changed. *Cis*-action refers to the direct action of lncRNA on its neighbouring genes, so mRNAs within 100 kb upstream and downstream of lncRNA were extracted as *cis*-target genes of lncRNA. *Trans*-action means that the lncRNA can regulate gene expression from a distance, so mRNAs with correlation coefficients > 0.9 or <−0.9 with this lncRNA were selected as *trans* target genes. The *cis* target genes of lncRNAs were used with the bedtools window software with “-l 100,000” and “-r 100,000” parameters [54]; the *trans* target genes of lncRNAs were used with the R package Hmisc with correlation coefficients > 0.9 or <−0.9 and *p*-adjust < 0.05. Seven AS types were obtained via SUPPA software with default parameters [55].

### 4.5. Transcriptome Expression Analysis

The raw read counts were evaluated by featureCounts software and converted to *TPM* using an in-house R script [56], and the DEGs were analysed using DESeq2 software with an FDR ≤ 0.05 and a |log_2_(FoldChange)| ≥ 2 [57].

The τ score of all genes was based on the method of Yanai et al. [58,59]. The τ score was calculated using an in-house Python script, and genes with a score > 0.95 were thought to have a high potential for stage-specific expression in fruit development.

The R package Mclust was used to perform the time series analysis [60]. First, a mean *TPM* value was calculated across the three biological replicates of the four developmental stages and converted to z-scores, and the genes with a mean *TPM* ≥ 1 in at least one stage were retained for subsequent analysis. Genes in the top 10% of expression variance were used with the function mclustBIC to calculate the most suitable cluster number. Based on the results, the most suitable model was “VVV”, and the most suitable cluster number was 16.

### 4.6. qRT-PCR

Primers for qRT-PCR were designed with Primer-BLAST (https://www.ncbi.nlm.nih.gov/tools/primer-blast/index.cgi?LINK_LOC=BlastHome) (accessed on 12 September 2021), and their specificity was verified by PCR. qRT-PCR assays were conducted in triplicate using FastStart Universal SYBR Green Master Mix (Roche, Indianapolis, IN, USA) with actin as the reference on a ViiA7 Real-Time PCR system (ABI, Carlsbad, CA, USA). Relative expression levels were estimated via the 2^−^^△△Ct^ method according to the threshold of the PCR cycle.

## 5. Conclusions

By constructing the Iso-seq library, we obtained 46,715 high-quality full-length isoforms for improving the annotation of the *G. jasminoides* genome and mining novel genes. Compared with the annotation information deposited in the database, we identified 143 novel genes in the *G. jasminoides* genome that could transcribe 147 transcripts, while we also identified 5083 transcripts derived from 4500 known genes, and that information contributes to the integrity of the genome annotation of *G. jasminoides*. LncRNAs are non-coding RNAs that are more than 200 nt in length and do not have coding potential. We identified 960 lncRNAs that were considered by CPC2, CNKI, and LGC as not having coding potential, which could target 13,484 genes in *cis* and 12,711 genes in *trans*, thus forming a complex regulatory network. AS, an important mechanism regulating gene expression, is prevalent in eukaryotes. A total of 6052 AS events of seven types involving 3823 genes were identified using SUPPA software. Some DEGs and genes with high potential for stage-specific expression in fruit development were significantly enriched in pathways such as “diterpenoid biosynthesis”. The product of the diterpenoid pathway is geranylgeranyl diphosphate, which is also a precursor of crocin, suggesting that these genes play a role in crocin biosynthesis.

In the crocin biosynthesis pathway, 19, 5, and 22 genes were involved in the upstream, midstream, and downstream pathways, respectively. *ALDH* is a large family, and fourteen different ALDH protein families have been discovered in plants. The dehydrogenation of the crocin pathway, which catalyses crocetin dialdehyde to produce crocetin, was performed by *ALDH*. In this study, the expression level of 12 ALDH genes increased gradually from the JUL stage and reached a maximum in NOV. UGT is the last enzyme that catalyses the conversion of crocetin to crocins. The expression patterns of five *UGT* genes were found to be consistent with the observed crocin accumulation. These findings contributed to our understanding of crocin biosynthesis and accumulation in *G. jasminoides*.

## 6. Future Directions

In this study, we preliminarily investigated the protein-coding genes and long non-coding RNAs involved in crocin I biosynthesis, but there are still some key questions to be addressed. First: the contribution of the MEP and MVP pathways to crocin I biosynthesis. There are two pathways for the upstream biosynthesis of crocin I, the MEP and MVA pathways, and the products of both IPP and its isomer DMAPP are precursors of crocin I. However, the contribution of MEP and MVA pathways to crocin I biosynthesis is still not known and has been poorly studied in other species. Second: Environmental effects on crocin I biosynthesis. The environment surrounding the plant can have a tremendous impact on its growth. In an unfavourable environment, such as drought, heat, and herbivory, will *G. gardenia* still biosynthesise crocin I, which is important for human health? Third: Are there other non-coding regulators in the crocin I biosynthesis pathway in *G. gardenia*, such as miRNA, siRNA, and circRNA?

## Supplementary Materials

The following supporting information can be downloaded at: https://www.mdpi.com/article/10.3390/ijms23116321/s1.
