# Supplementary figures and images for: Integrated SMRT and Illumina Sequencing Provide New Insights into Crocin Biosynthesis of Gardenia jasminoides
Source: Int J Mol Sci. 2022 Jun 5;23(11):6321. doi: 10.3390/ijms23116321 (PMC9181021; doi:10.3390/ijms23116321)

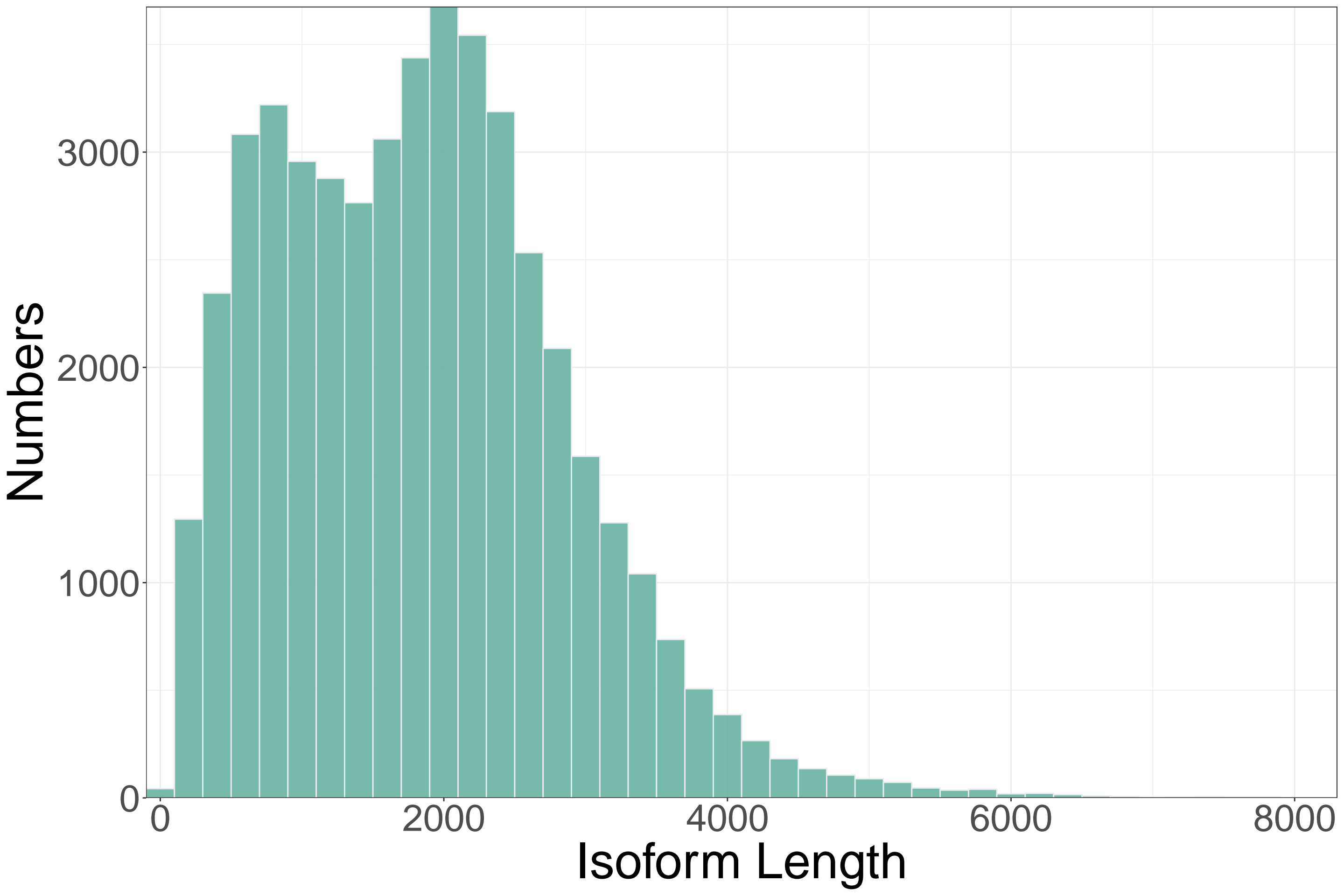

Supplement: Supplementary file 1 [file ijms-23-06321-s001.zip › Figure S1.pdf]

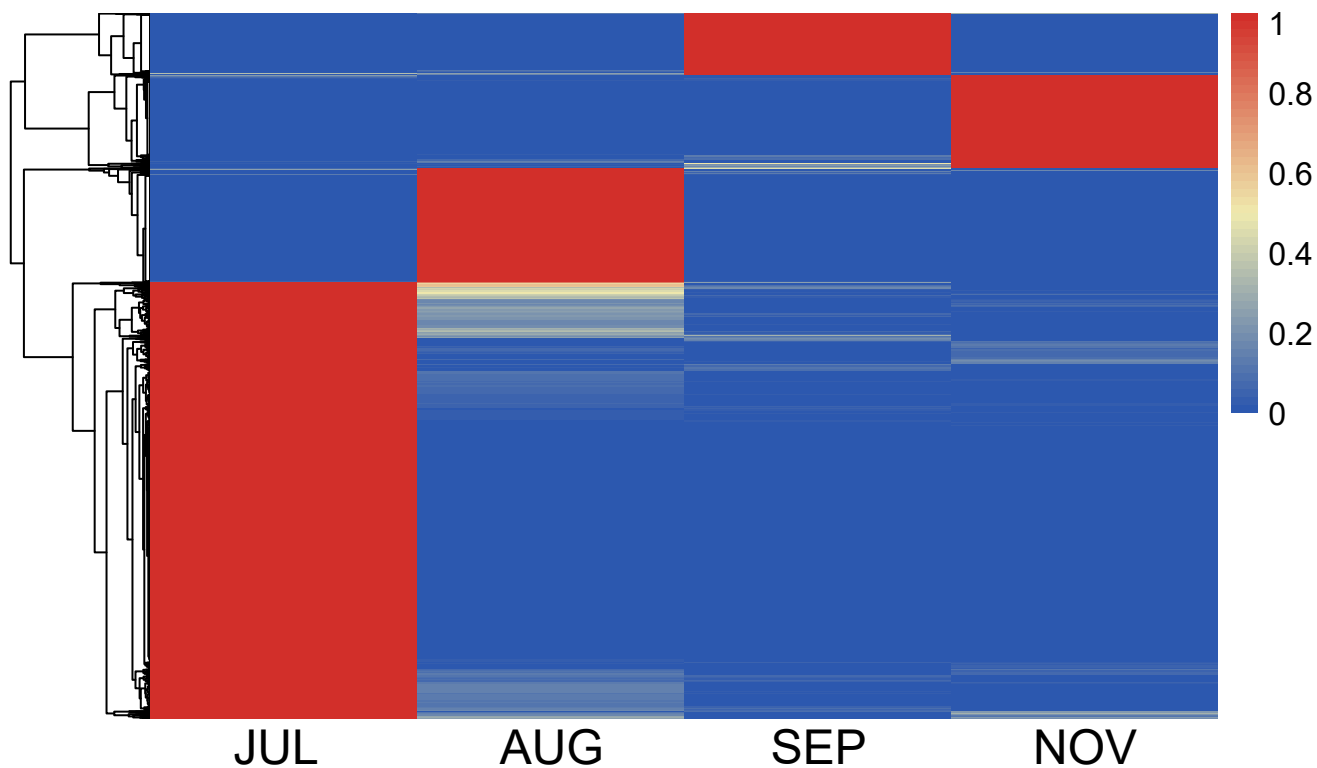

Supplement: Supplementary file 1 [file ijms-23-06321-s001.zip › Figure S10.pdf]

# Statistics of Pathway Enrichment

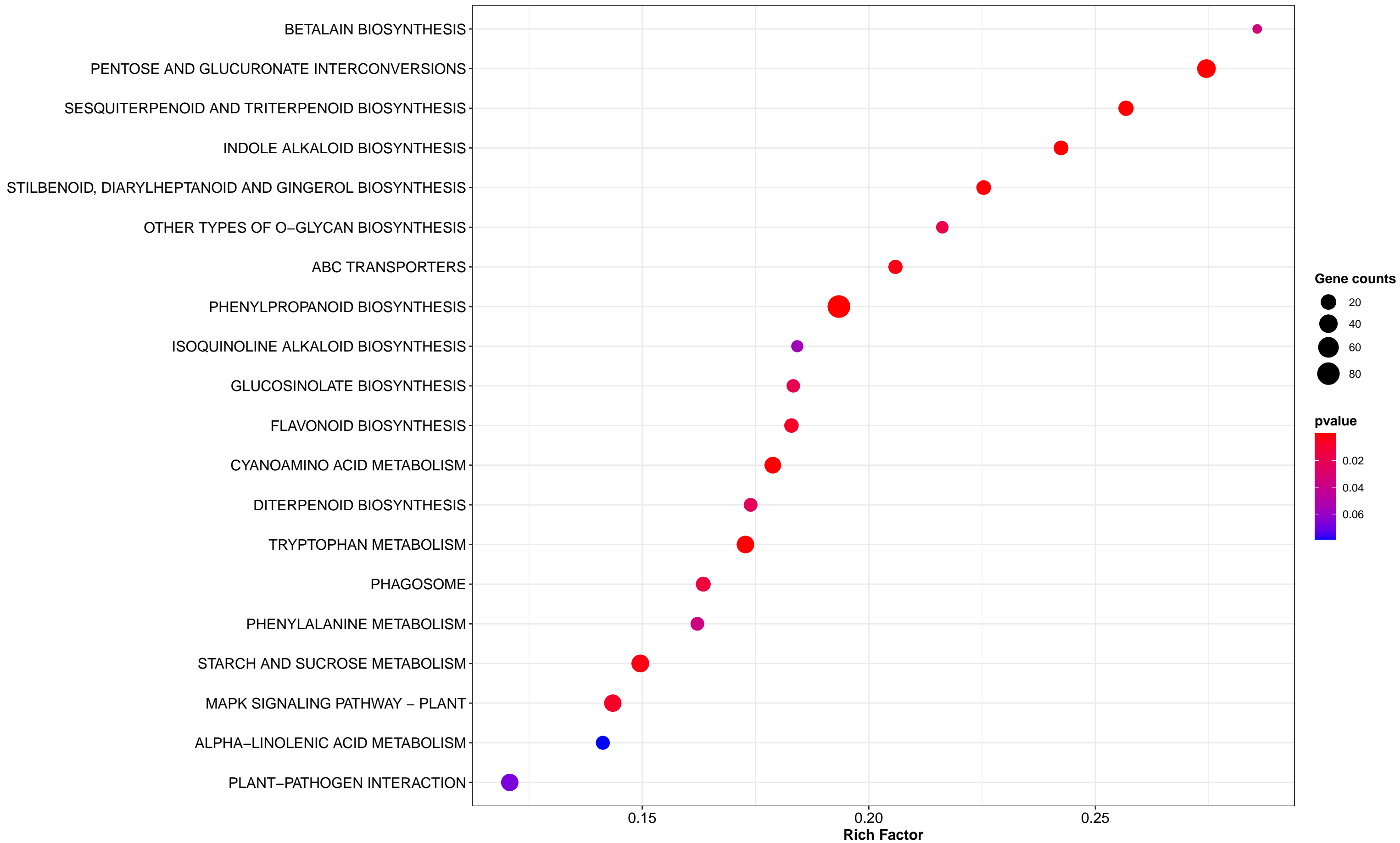

Supplement: Supplementary file 1 [file ijms-23-06321-s001.zip › Figure S11.pdf]

BIC

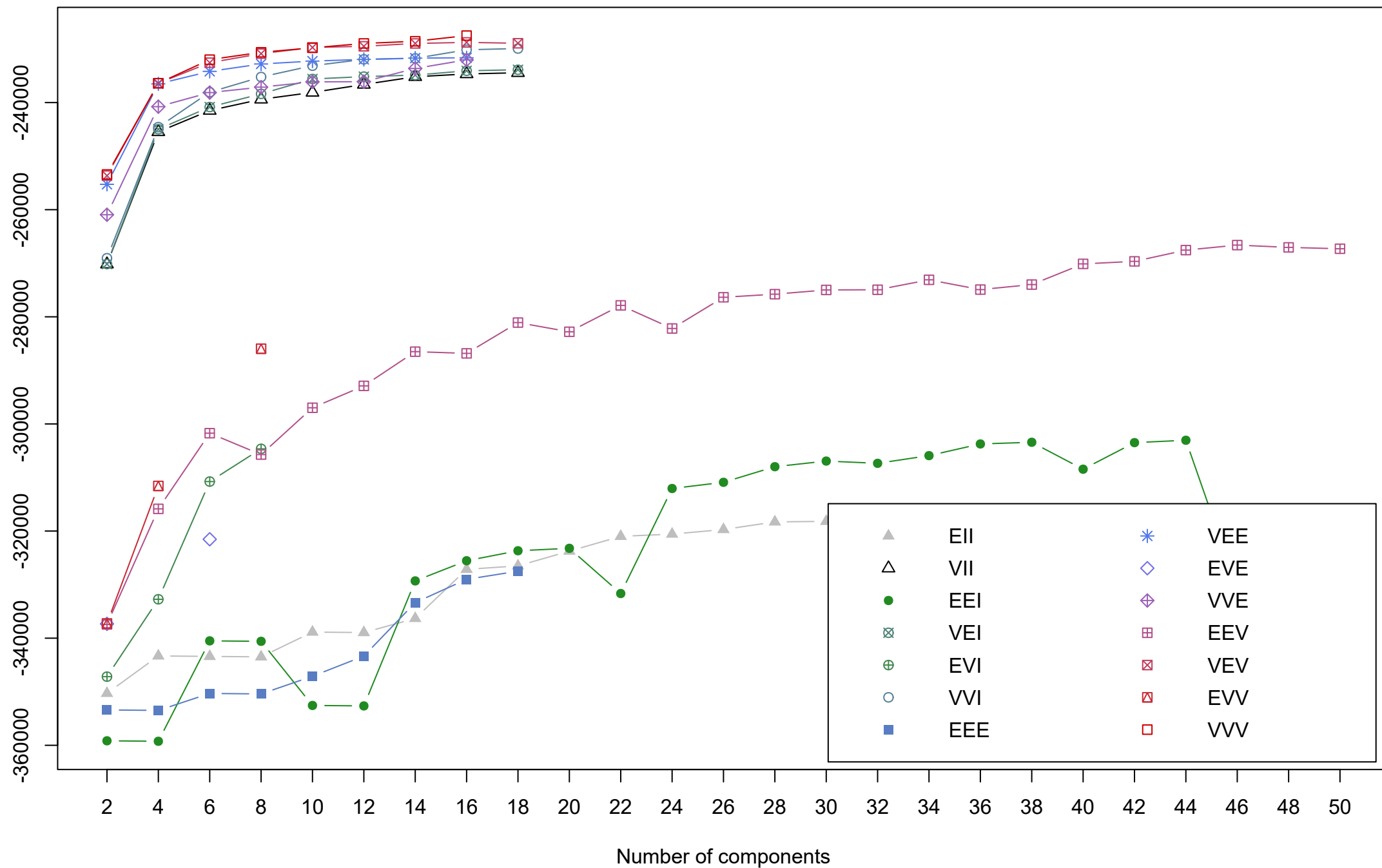

Supplement: Supplementary file 1 [file ijms-23-06321-s001.zip › Figure S12.pdf]

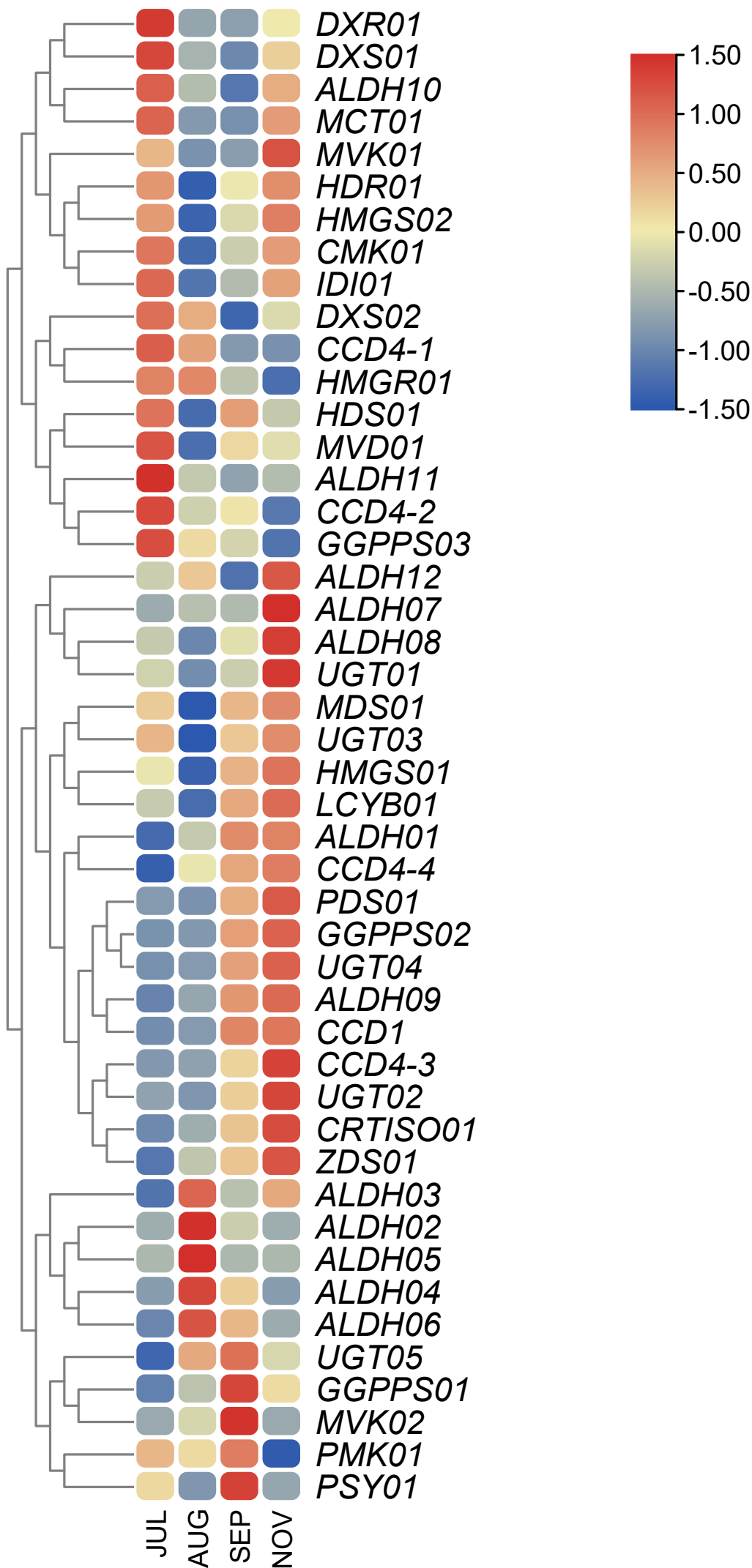

Supplement: Supplementary file 1 [file ijms-23-06321-s001.zip › Figure S13.pdf]

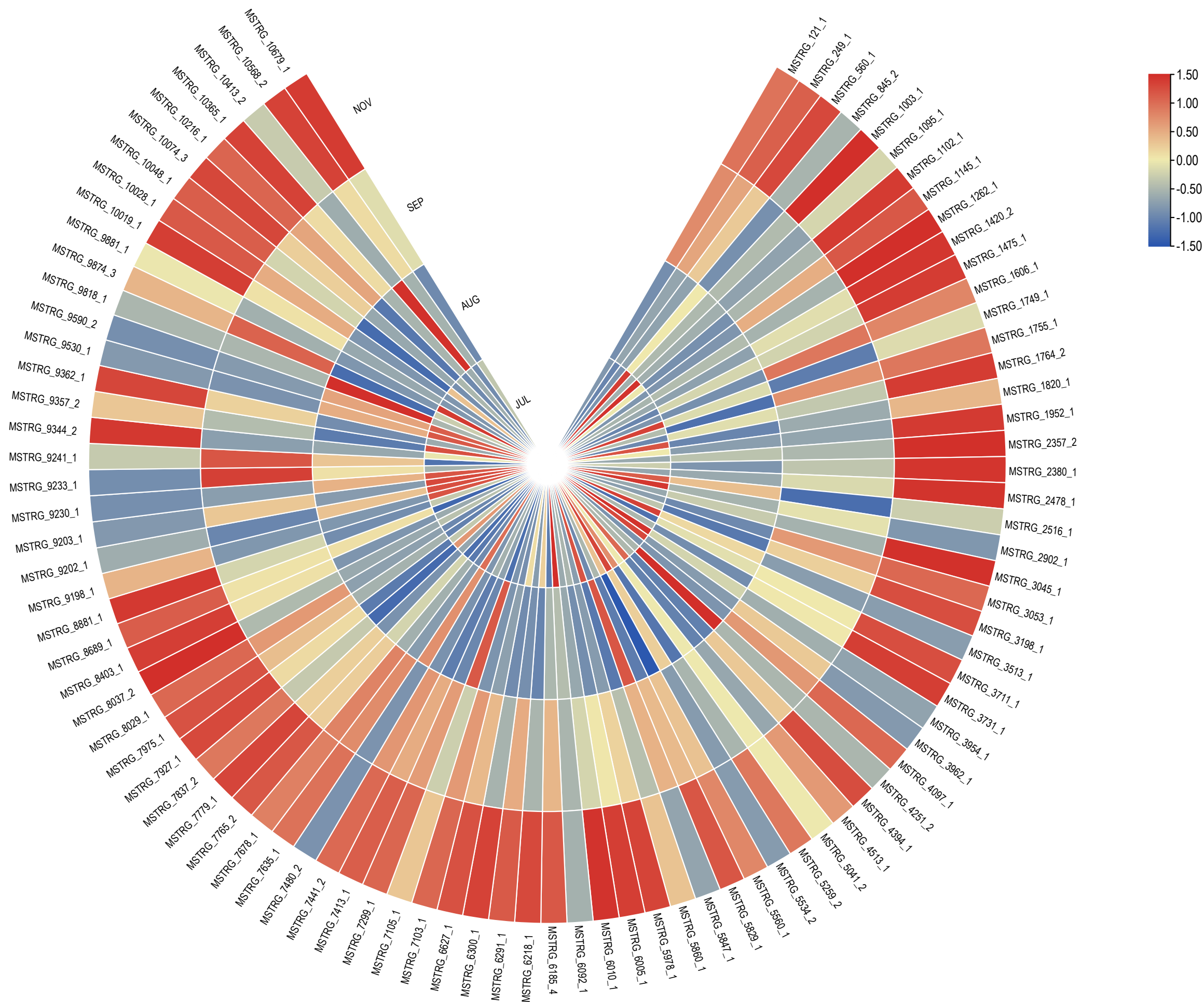

Supplement: Supplementary file 1 [file ijms-23-06321-s001.zip › Figure S14.pdf]

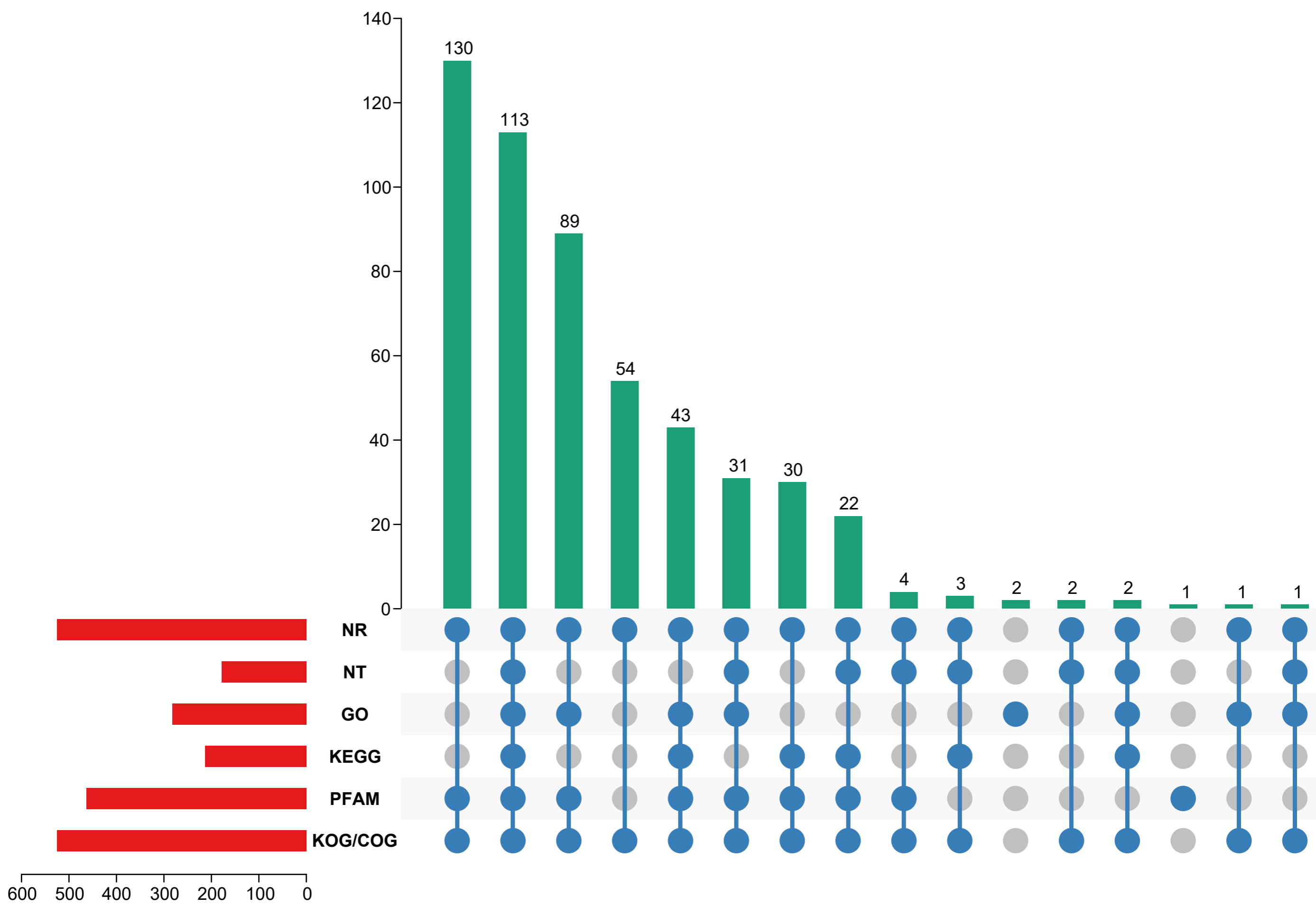

Supplement: Supplementary file 1 [file ijms-23-06321-s001.zip › Figure S2.pdf]

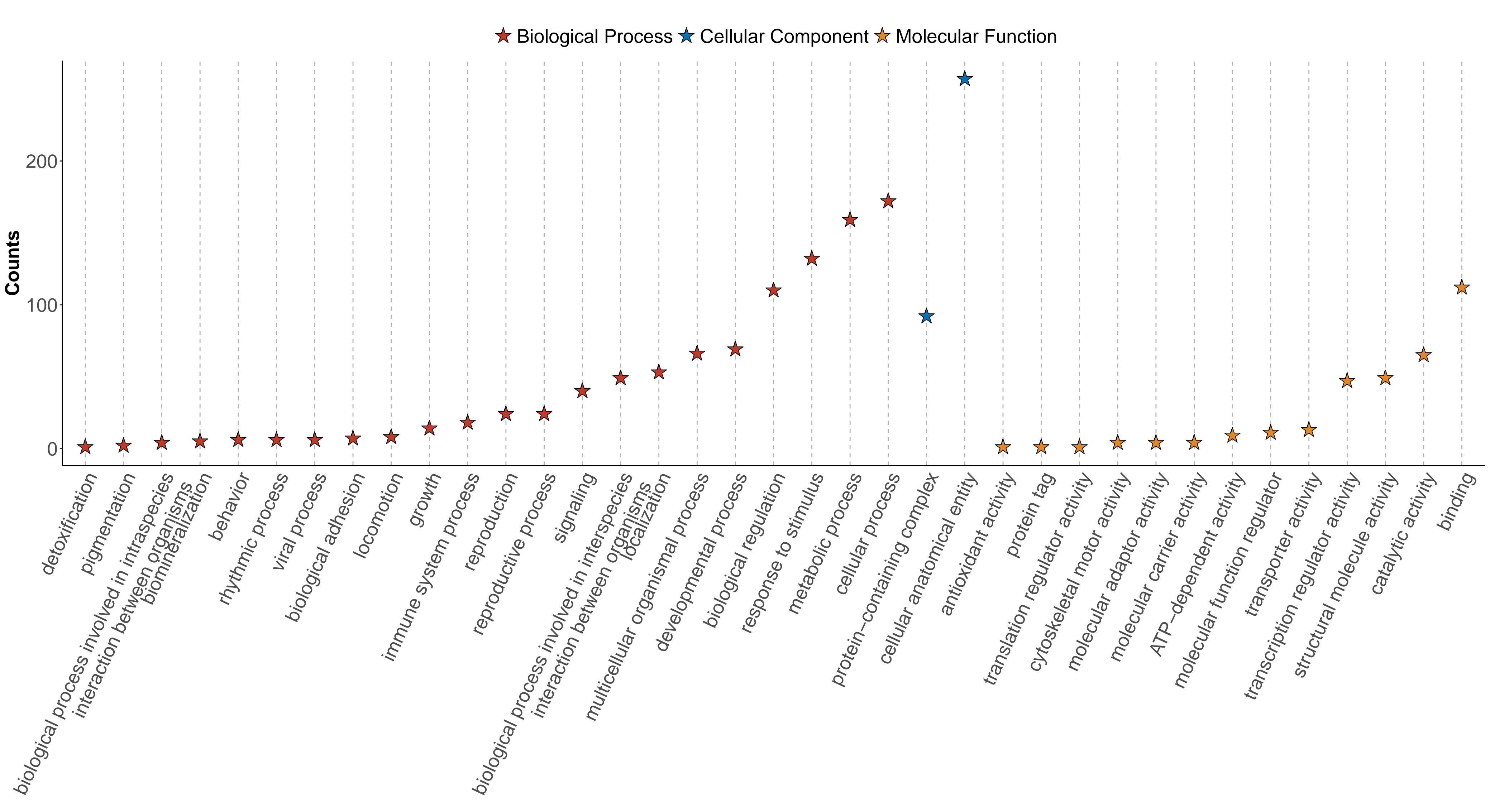

Supplement: Supplementary file 1 [file ijms-23-06321-s001.zip › Figure S3.pdf]

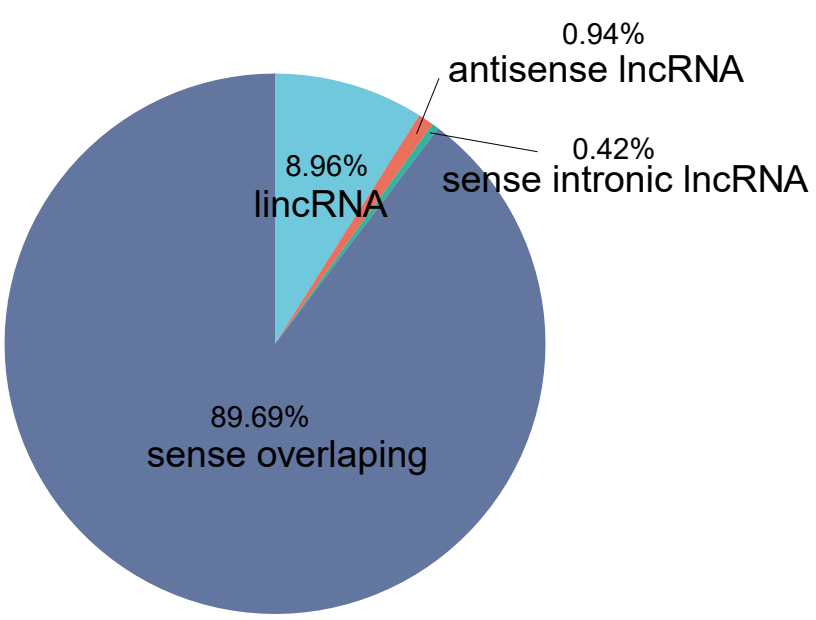

Supplement: Supplementary file 1 [file ijms-23-06321-s001.zip › Figure S4.pdf]

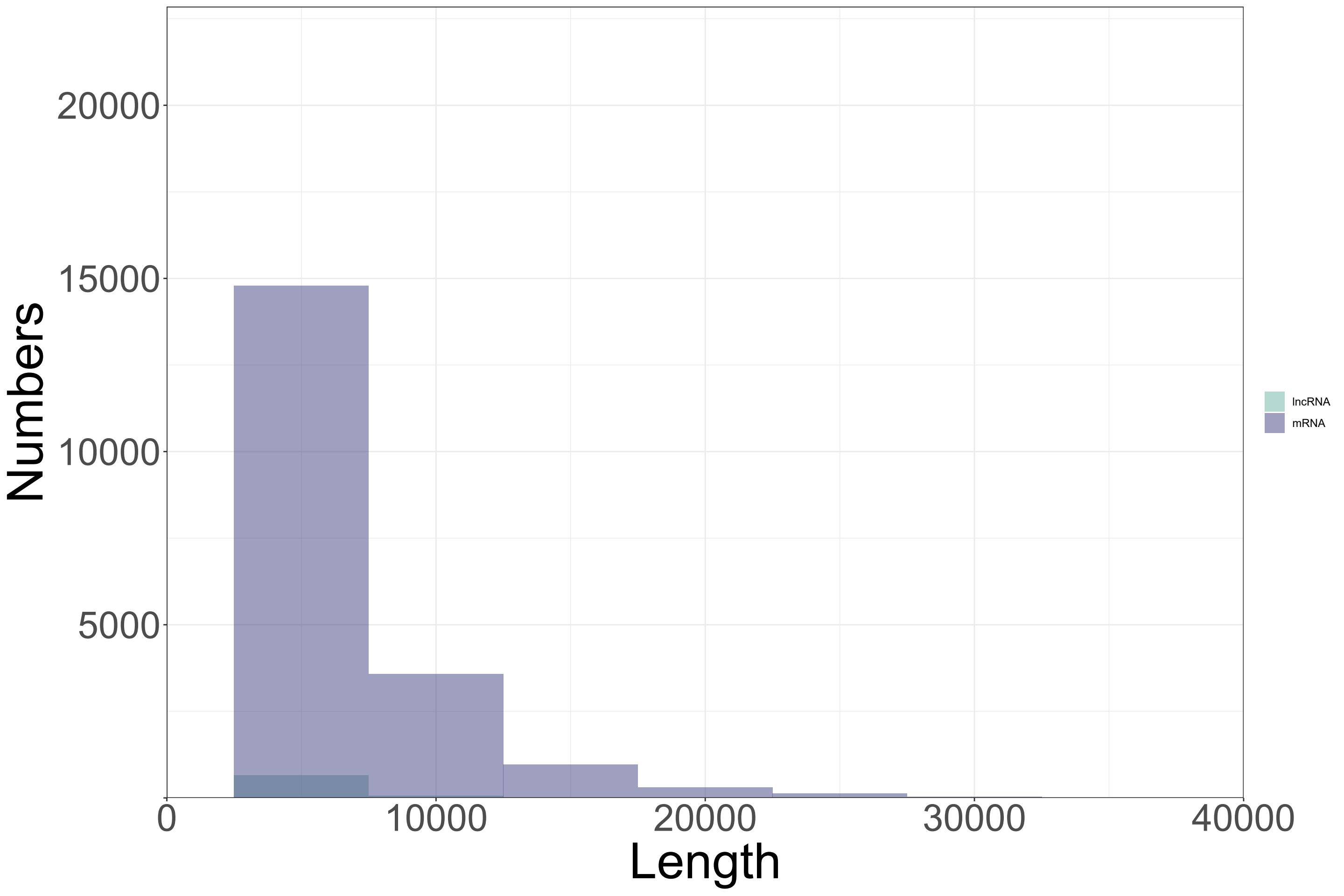

Supplement: Supplementary file 1 [file ijms-23-06321-s001.zip › Figure S5.pdf]

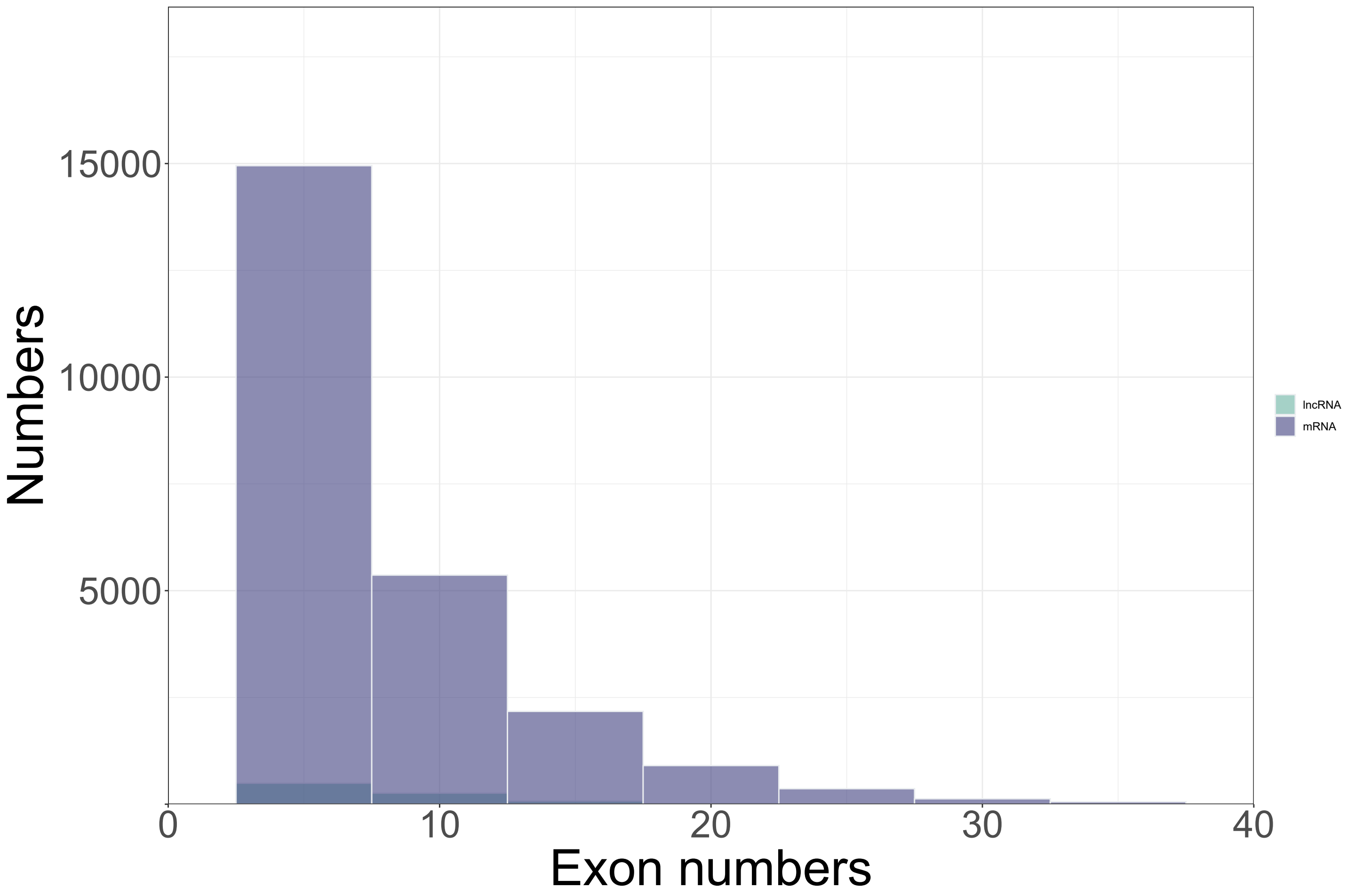

Supplement: Supplementary file 1 [file ijms-23-06321-s001.zip › Figure S6.pdf]

# Statistics of Pathway Enrichment

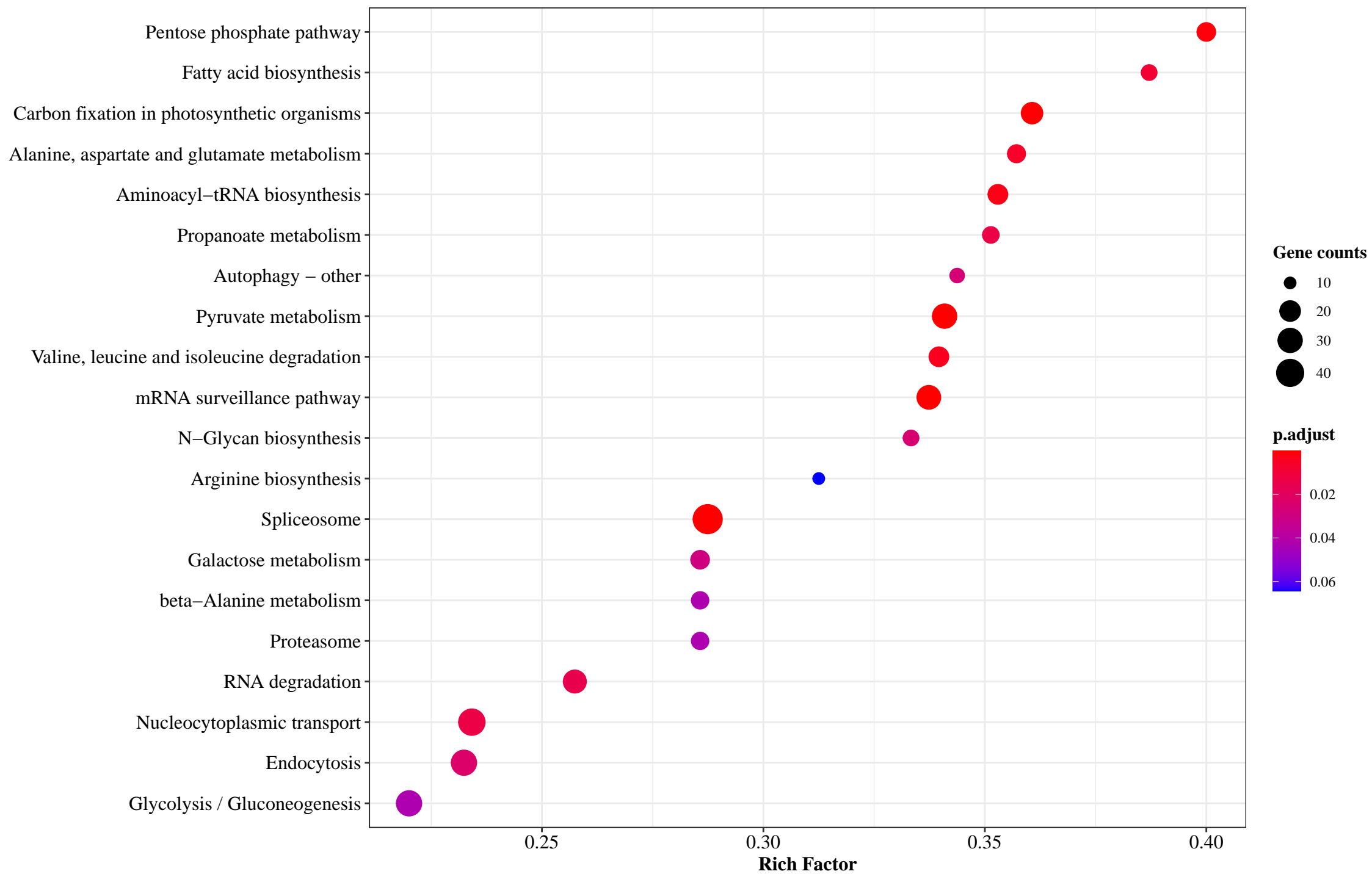

Supplement: Supplementary file 1 [file ijms-23-06321-s001.zip › Figure S7.pdf]

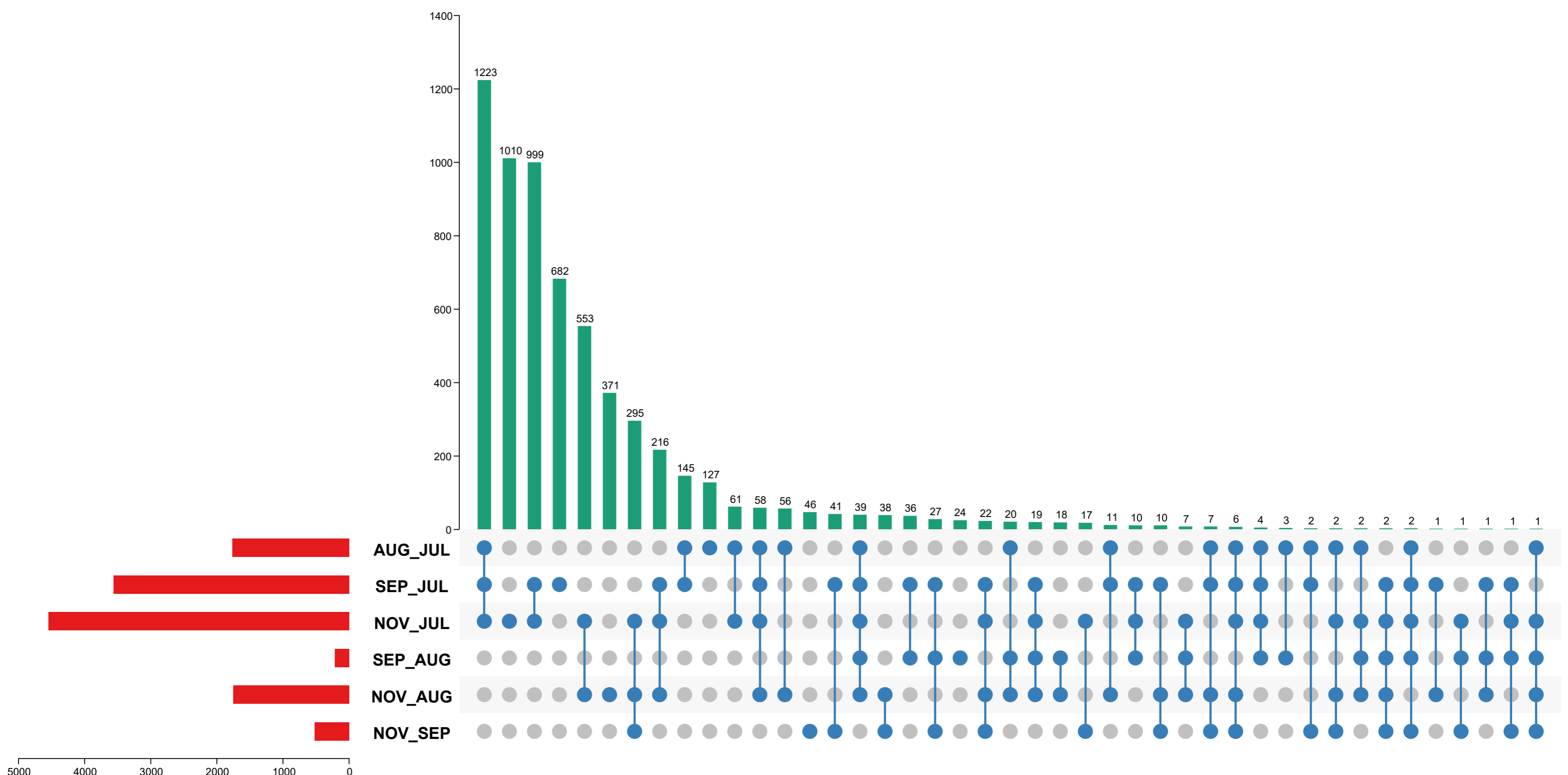

Supplement: Supplementary file 1 [file ijms-23-06321-s001.zip › Figure S8.pdf]

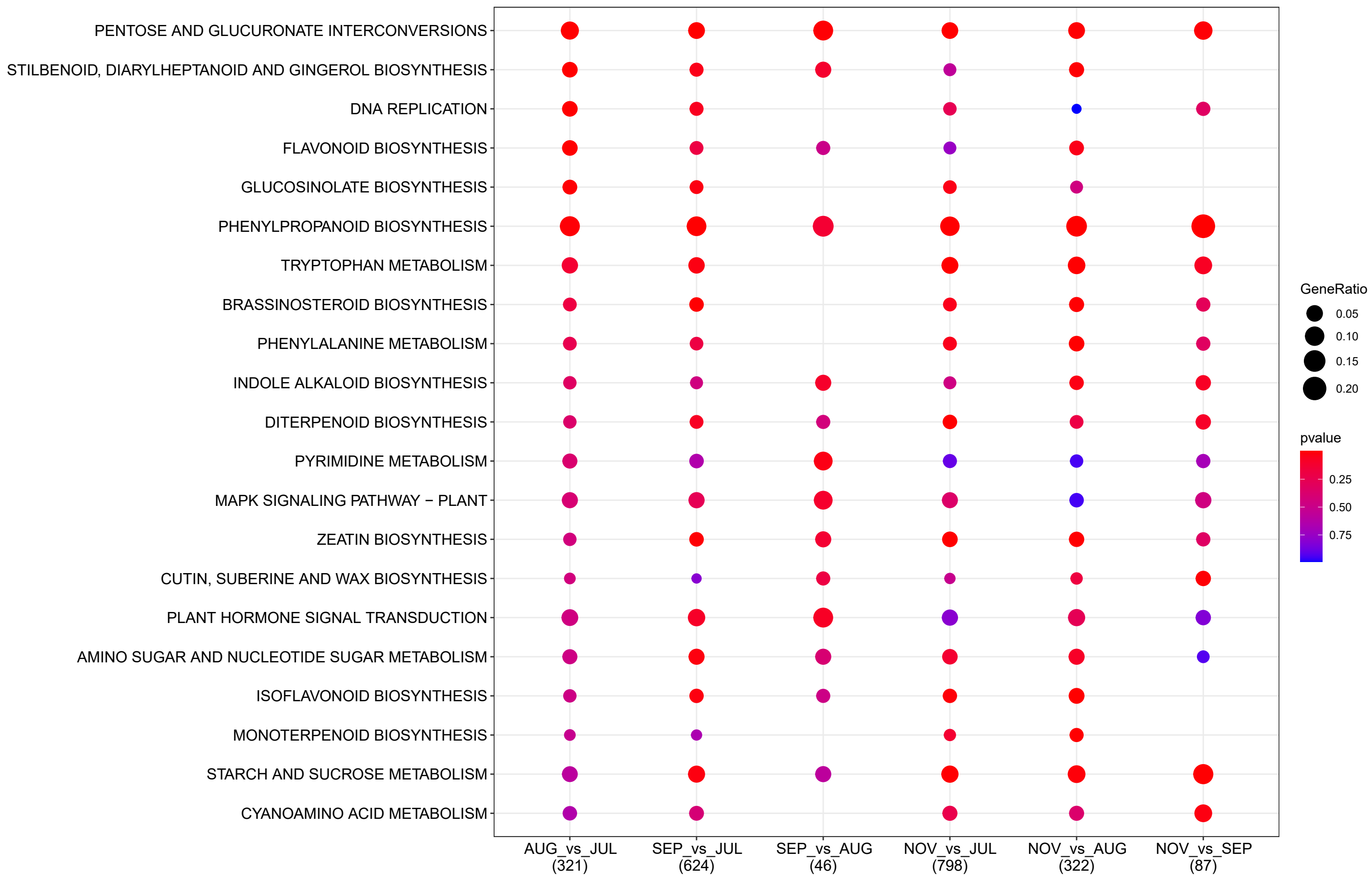

Supplement: Supplementary file 1 [file ijms-23-06321-s001.zip › Figure S9.pdf]
